# Noncanonical role of singleminded-2s in mitochondrial respiratory chain formation in breast cancer

**DOI:** 10.1038/s12276-023-00996-0

**Published:** 2023-05-01

**Authors:** Steven W. Wall, Lilia Sanchez, Kelly Scribner Tuttle, Scott J. Pearson, Shivatheja Soma, Garhett L. Wyatt, Hannah N. Carter, Ramsey M. Jenschke, Lin Tan, Sara A. Martinez, Philip L. Lorenzi, Vishal M. Gohil, Monique Rijnkels, Weston W. Porter

**Affiliations:** 1 Department of Veterinary Physiology and Pharmacology, School of Veterinary Medicine, Texas A&M University, College Station, TX 77843 USA; 2 Center for Toxicology and Environmental Health, North Little Rock, AR 72118 USA; 3 Department of Biochemistry & Biophysics, Texas A&M University, College Station, TX 77843 USA; 4 Metabolomics Core Facility, Department of Bioinformatics and Computational Biology, The University of Texas MD Anderson Cancer Center, Houston, TX 77054 USA; 5 Department of Veterinary Integrative Biosciences, School of Veterinary Medicine, Texas A&M University, College Station, TX 77843 USA

**Keywords:** Breast cancer, Energy metabolism

## Abstract

Dysregulation of cellular metabolism is a hallmark of breast cancer progression and is associated with metastasis and therapeutic resistance. Here, we show that the breast tumor suppressor gene *SIM2* promotes mitochondrial oxidative phosphorylation (OXPHOS) using breast cancer cell line models. Mechanistically, we found that SIM2s functions not as a transcription factor but localizes to mitochondria and directly interacts with the mitochondrial respiratory chain (MRC) to facilitate functional supercomplex (SC) formation. Loss of *SIM2s* expression disrupts SC formation through destabilization of MRC Complex III, leading to inhibition of electron transport, although Complex I (CI) activity is retained. A metabolomic analysis showed that knockout of *SIM2s* leads to a compensatory increase in ATP production through glycolysis and accelerated glutamine-driven TCA cycle production of NADH, creating a favorable environment for high cell proliferation. Our findings indicate that SIM2s is a novel stabilizing factor required for SC assembly, providing insight into the impact of the MRC on metabolic adaptation and breast cancer progression.

## Breast cancer: Possible route to blocking cancer progression

Insights into the stabilising role of a metabolism-related protein could inform treatments that prevent breast cancer cells from proliferating. Oxidative phosphorylation is a metabolic process wherein cells use enzymes to metabolize nutrients, generating energy to produce adenosine triphosphate (ATP). Tumour cells actively reprogram such metabolic pathways to promote cancer progression. Weston Porter at Texas A&M University, College Station, USA, and co-workers examined the roles of the breast cancer suppressor gene singleminded-2s (*SIM2s*), in breast cell lines. In normal cells, SIM2s localises to the mitochondria and interacts with the mitochondrial respiratory chain (the complex involved in generating ATP) to create a supercomplex that promotes oxidative phosphorylation. Loss of *SIM2s* in breast cancer destabilizes supercomplex formation, resulting in compensatory ATP production via another pathway controlled by the tumour, feeding cancer progression.

## Introduction

Metabolic reprogramming has been established as a hallmark of tumor cells^1–3^. First described by Otto Warburg, tumor cells preferentiallly utilize glycolysis while oxidative phosphorylation (OXPHOS) is downregulated, even in the presence of abundant oxygen^4–6^. The glycolytic pathway confers a selective growth advantage and promotes metastasis by inducing the production of metabolic intermediates and ATP for cell proliferation without generating ROS^5–8^. Moreover, in most cancers, glutamine (Gln) metabolism is activated, re-establishing the levels of tricarboxylic acid (TCA) cycle intermediates that are consumed in the synthesis of nucleic acids, amino acids, and glutathione, promoting cell survival and proliferation^4,9–11^. Previous work has shown that altered metabolism in breast cancer is associated with increased tumor progression and therapeutic resistance, supporting a role for altered metabolism in malignant transformation^12–17^.

Although the majority of these reprogramming events have been associated with several oncogene pathways, including PI3K/AKT and hypoxia-inducible factor (HIF1α), metabolic alterations that inhibit OXPHOS and promote cancer progression can be linked to defects in mitochondrial respiration chain (MRC) assembly. The MRC is composed of four multisubunit enzyme complexes and two mobile electron carriers that aggregate in varying stoichiometric ratios to form macromolecular structures commonly known as supercomplexes (SCs)^18,19^. The majority of mammalian SCs comprise Complex I (CI) (i.e., SC I + III_2_, SC I + III_2_ + IV_1-4_ [respirasomes], and SC I_2_ + III2 + IV_2_ [megacomplex]), except for SC III_2_ + IV_1-2_^20,21^. SCs are formed simultaneously with individual MRC complexes, and their functions and formation mechanisms are still not clear^22–27^. Recent studies have shown that accessory subunits of individual complexes are critical for facilitating the stabilization of SCs, and changes in their expression can result in cancer progression^28–32^. Moreover, a recently proposed cooperativity model of MRC formation suggests that complex subunits and subassemblies can directly form SCs, suggesting that fully formed MRC complexes are not required for SC formation^33^.

We have previously shown that the short splice variant of the bHLH/PAS transcription factor Singleminded-2 (SIM2s) plays an important role in normal mammary gland development and tumor differentiation^34–40^. Human *SIM2* was initially identified by positional cloning around the Down Syndrome (DS) critical region of chromosome 21 and is amplified in DS patients and mouse models^41,42^. *SIM2s* expression is lost in primary breast tumors but not in normal mammary glands^35,38^, and loss of *SIM2s* expression is associated with an epithelial–mesenchymal transition (EMT). Re-establishment of *SIM2s* expression in breast cancer cell lines and xenografts inhibits cell proliferation and metastasis while promoting differentiation^35,38^. Additionally, we identified regulatory interaction of SIM2s with BRCA1-dependent homologous recombination (HR), a pathway critical to maintaining genome stability and preventing cancer^43,44^. In this study, we investigated the role of SIM2s in metabolism by knocking out and overexpressing *SIM2s* in breast cancer cell lines. We show that SIM2s localizes to the mitochondria and interacts with MRC subunits to promote OXPHOS, whereas loss of *SIM2s* disrupts SC formation, leading to metabolic reprogramming through the activation of glycolysis and Gln metabolism. This work provides important insight into the impact of MRCs on cancer progression and provides novel targets for breast cancer therapies.

## Methods

### Cell culture

MCF7 and SUM159 cell lines (ATCC) were maintained in high-glucose (25 mM) DMEM with (1 mM) sodium pyruvate and (4 mM) L-glutamine (Thermo Fisher Scientific, Cat. 11995040) supplemented with 10% fetal bovine serum (R&D Systems) and 1% penicillin‒streptomycin (Thermo Fisher Scientific) at 37 °C with 5% CO_2_. The medium used for low glucose experiments comprised glucose, glutamine, sodium pyruvate, and phenol red free DMEM (Thermo Fisher Scientific, Cat. A1443011) supplemented with (1 mM) sodium pyruvate (Thermo Fisher Scientific), (4 mM) L-glutamine (Thermo Fisher Scientific), and D-glucose (Sigma‒Aldrich).

### Plasmids and cell line generation

*SIM2s-FLAG* SUM159 and *shSIM2* MCF7 cells were generated as previously described^36,44^. The CRISPR/Cas9 *SIM2* KO vector (pLV[2CRISPR]-hCas9:T2A:Puro-U6 > hSIM2[gRNA#1]-U6 > hSIM2[gRNA#2]) was designed with two gRNA sequences targeting exon 1 in h*SIM2* (gRNA#1 5’-TCCGCGCTACTTCCTCTTCA-3’; gRNA#2 5’-ATGCGCGCCGTCTTCCCCGA-3’) and was generated and transformed into Stbl3 competent cells by VectorBuilder.

Plasmid DNA was isolated using a ZymoPURE Plasmid DNA Isolation Kit (Zymo Research). Lentiviral transduction was performed as previously described, and cells were selected with 2 μg/mL puromycin (Sigma‒Aldrich) for 7 days or after five passages.

### siRNA transfections

Silencer Negative Control No. 1 siRNA (cat. AM4611) and two predesigned silencer siRNAs against UQCRC2 (cat. AM16708; item no. 104034[CIII.1] and 138976[CIII.2]) were purchased from Thermo Fisher Scientific (Cat. No. AM16708). The siRNA oligos were transfected into MCF7 cells at a final siRNA concentration of 10 nM. Lipofectamine RNAiMAX transfection reagent was used to perform reverse transfection according to the manufacturer’s protocol. Cells were collected 48 h after transfection.

### Immunoblotting

Cultured cells were washed with cold PBS and lysed in high-salt lysis buffer (50 mM HEPES, 500 mM NaCl, 1.5 mM EDTA, 10% glycerol, and 1% Triton X-100 at pH 7.5) supplemented with 1 mM Na_3_VO_4_, 1 mM cOmplete ULTRA Tablets, Mini, EDTA-free EASYpack (Roche), and 100 nM MG-132. Protein concentration was calculated using a DC Protein Assay Kit (Bio--Rad). Equivalent amounts of protein were mixed with 6X Laemmli buffer (250 mM Tris-HCl, 8% SDS, 40% glycerol, and 0.4 M dithiothreitol, pH 6.8) and incubated at 95 °C for 5 mins prior to loading onto 8%, 10%, 12%, or 15% SDS‒PAGE gels (Bio–Rad). Gels were run using PowerPak2000 (Bio–Rad) until immediately before the dye front reached the bottom. Immunoblotting was performed using Mini Trans-Blot Cell (Bio–Rad), and proteins were transferred onto Immun-Blot PVDF membranes (Bio–Rad). The membranes were blocked in 5% instant nonfat dry milk (Great Value) in TBST for 1 h at room temperature with agitation. Primary antibodies were diluted as described in Supplementary Table 2 in TBST and incubated overnight at 4 °C with agitation. The membranes were washed with TBST, and HRP-linked secondary antibodies (Cell Signaling) were diluted 1:5000 in TBST and incubated with the membranes for 1 h at room temperature with agitation. The membranes were washed in TBST, and bands were visualized using ProSignal Pico ECL spray (Prometheus) and digitized with a ChemiDoc MP Imaging System (Bio–Rad). Band intensities were measured using Fiji and normalized first to the loading control and then to the control sample.

### RNA isolation and real-time PCR

RNA isolation and real-time PCR were performed and analyzed as described^45^. Briefly, RNA was isolated using a High Pure RNA Isolation Kit (Roche), cDNA was generated using an iScript cDNA Synthesis Kit (Bio-Rad), and real-time PCR was run using iTaq Universal SYBR (Bio–Rad) on a C1000 thermocycler with a CFX384 real-time system (Bio–Rad). Primers sequences for the genes analyzed can be found in Supplementary Table 3.

### Proliferation assay

Cells were counted using a Cellometer Auto 100 Bright Field Cell Counter (Nexcelom), and 10,000 cells were plated in triplicate. Cells were allowed to adhere overnight, and control or glucose-depleted medium was added. Fresh medium was added after two days of culture, and the cells were collected after three complete days in culture. The cells were harvested using trypsin treatment for detachment and then pelleted. Pellets were resuspended in growth medium, and cells were counted in triplicate.

### Analysis of polar metabolites by LC/IC-HRMS

Extracts were prepared and analyzed by high-resolution mass spectrometry (HRMS) to determine the consumption level of glucose and glutamine carbon in glycolysis, the tricarboxylic acid (TCA) cycle, and amino acid synthesis. MCF7 cells in log-phase growth were seeded in 10-cm dishes. The cells were incubated in fresh RPMI medium (US Biologicals) containing 11.1 mM [1,2-^13^C2]-glucose and 2 mM [^13^C5]-glutamine for 4 and 24 h, respectively. At the indicated time points, cells were quickly washed with ice-cold deionized water, and then, metabolites were extracted using cold 80/20 (v/v) methanol/water with 0.1% ammonium hydroxide. Samples were centrifuged at 17,000 × *g* for 5 min at 4 °C, and supernatants were transferred to clean tubes, followed by evaporation to dryness under nitrogen. Samples were reconstituted in deionized water, and then 5 μL was injected into a Thermo Scientific Dionex ICS-5000+ capillary ion chromatography (IC) system containing a Thermo IonPac AS11 250 × 2-mm × 4-μm column. The IC flow rate was 360 μL/min (at 30 °C), and the gradient conditions were as follows: initial 1 mM KOH, increased to 35 mM at 25 min, then increased to 99 mM at 39 min, and held at 99 mM for 10 min. The total run time was 50 min. To increase sensitivity via desolvation, methanol was added via an external pump and combined with the eluent via a low dead volume mixing tee. Data were acquired using a Thermo Orbitrap Fusion Tribrid Mass Spectrometer in negative ESI mode.

For amino acid analysis, samples were diluted in 90/10 acetonitrile/water containing 1% formic acid, and then, 15 μL of prepared sample was injected into a Thermo Vanquish liquid chromatography (LC) system containing an Imtakt Intrada Amino Acid 2.1 × 150-mm column with a 3-µm particle size. Mobile phase A (MPA) was acetonitrile with 0.1% formic acid. Mobile phase B (MPB) was 50 mM ammonium formate. The flow rate was 300 μL/min (at 35 °C), and the gradient conditions were as follows: initial 15% MPB, increased to 30% MPB at 20 min, then increased to 95% MPB at 30 min, held at 95% MPB for 10 min, returned to initial conditions and equilibrated for 10 min. The total run time was 50 min. Data were acquired using a Thermo Orbitrap Fusion Tribrid mass spectrometer under positive ESI mode at a resolution of 240,000. Then, raw files were imported into Thermo Trace Finder software for final analysis. The fractional abundance (or mass distribution vector; MDV) of each isotopolog was calculated as the peak area of the corresponding isotopolog normalized on the basis of the sum of all isotopolog areas^46^. Isotopic enrichment is defined as $\mathop {\sum}\nolimits_{i = 1}^N {\quad i \cdot M_i}$, where *M*_*i*_ is the MDV, and *N* is the total number of carbon atoms in a molecule. The relative abundance of each metabolite was normalized on the basis of the total peak intensity.

### Seahorse assay

Cellular respiration was analyzed on a Seahorse XFe96 Bioanalyzer (Agilent Technologies). The Seahorse XF Cell Mito Stress Test Kit (Agilent Technologies) was used to determine the oxygen consumption rate (OCR) and extracellular acidification rate (ECAR). The experiment was performed according to the manufacturer’s protocol using Seahorse XF DMEM, pH 7.4, recommended concentrations of medium supplements and experimental drugs with one exception: FCCP was used at a final concentration of 0.5 μM. Cells were seeded at a density of 15,000 cells per well, and cell numbers were normalized either by staining cells with 20 μM Hoechst 33342 (Invitrogen) and measuring relative fluorescence (Ex. 350, Em. 461; area scan) on a SynergyH1 plate reader (BioTek) or by lysing cells using high-salt lysis buffer and measuring the relative protein concentrations using a DC protein assay kit (Bio–Rad) after the experiment. Mean OCR and ECAR values were compared on the basis of a minimum of ten replicates. ATP production rates were calculated using Wave software (Agilent).

### Cellular fractionation

Crude mitochondrial fractions were extracted by differential centrifugation, as previously described^47^. Briefly, a large cell pellet was lysed in RSB hypo buffer (10 mM NaCl, 1.5 mM MgCl_2_, 10 mM Tris-HCl, pH 7.5) on ice. Cell lysis was facilitated by the use of a Dounce homogenizer, and lysis was halted by the addition of MSH buffer (210 mM mannitol; 70 mM sucrose; 5 mM Tris-HCl; and 1 mM EDTA, pH 7.5). All buffers (RSB, MSH, GRO, and lysis) were supplemented with 1 mM Na_3_VO_4_ and 1 mM cOomplete ULTRA Tablets, Mini, EDTA-free EASYPack (Roche) to inhibit phosphatase and protease activity, respectively, and all centrifugation steps were performed in a prechilled (4 °C) centrifuge. Cell suspensions were centrifuged at 600 × *g* for 5 min, and the supernatant transferred to a new tube. The cell pellets containing unbroken cells and nuclei were saved for nuclei isolation. Supernatants were then centrifuged at 7000 × *g* for 10 min, and the pellet (mitochondrion-enriched fraction) was retained. Supernatants from this step were concentrated using Amicon Ultra centrifugal filters, 10 kD (Sigma Aldrich), to generate cytoplasmic fractions per the manufacturer’s instructions. The mitochondria-enriched fraction was resuspended in MSH buffer, and the centrifugation and wash steps were repeated. Finally, the suspension was spun at 10,000 × *g* for 10 min, and the supernatant was discarded. The crude mitochondrial pellet was resuspended in MSH buffer, assayed using a DC protein assay kit (Bio–Rad), and aliquoted in 50-μg increments for native applications. For non-native applications, the crude mitochondrial pellet was resuspended in high-salt lysis buffer (50 mM HEPES, 500 mM NaCl, 1.5 mM EDTA, 10% glycerol, and 1% Triton X-100 at pH 7.5) supplemented with 1 mM Na_3_VO_4_, 1 mM cOmplete ULTRA Tablets, Mini, EDTA-free EASYpack (Roche), and 100 nM MG-132. Nuclei were purified from discarded cell pellets by resuspension in GRO lysis buffer (2 mM MgCl_2_, 3 mM CaCl_2_, 0.5% NP-40, and 10 mM Tris-HCl, pH 7.4) with a Dounce homogenizer. Homogenates were filtered through a 40-μm cell strainer (VWR) and spun at 500 × *g* for 5 min. Nuclear pellets were resuspended in high-salt lysis buffer (50 mM HEPES, 500 mM NaCl, 1.5 mM EDTA, 10% glycerol, and 1% Triton X-100 at pH 7.5) supplemented with 1 mM Na_3_VO_4_, 1 mM cOmplete ULTRA Tablets, Mini, EDTA-free EASYpack (Roche), and 100 nM MG-132.

### Blue native polyacrylamide gel electrophoresis (BN-PAGE) and complexes I and IV in-gel activity

Samples were prepared by resuspending 50 μg of native mitochondrial pellets in 1× NativePAGE Sample Buffer (Invitrogen), digitonin (8 g/g or 16 g/g for BN-PAGE, 0.4% for complex I in-gel activity, and 1% for complex IV in-gel activity) (Invitrogen) assays, and water to a final volume of 20 μL and mixed by up and down pipetting. Samples were incubated on ice for 15 min to solubilize the mitochondrial proteins. The samples were then centrifuged in a precooled centrifuge (4 °C) for 30 min at 18,000 × *g*, and then, the supernatants were transferred to new tubes. Next, 0.5% G-250 Coomassie dye (Invitrogen) was added to the BN-PAGE samples, but this step was omitted for the in-gel activity samples.

For BN-PAGE, an XCell SureLock Mini-Cell (Invitrogen) with a NativePAGE Novex 3-12% Bis–Tris gel (Invitrogen) was established, and 1× NativePAGE anode buffer (1× NativePAGE running buffer), NativePAGE dark blue cathode buffer (1× NativePAGE running buffer and 1× cathode additive), and NativePAGE Light Blue Cathode buffer (1× NativePAGE running buffer and 0.1× Cathode Additive) were mixed according to the NativePAGE Novex Bis–Tris Gel System protocol. The wells of the gel were then filled with dark blue cathode buffer, samples and NativeMark unstained protein standard (Invitrogen). The inner chamber was filled with Dark Blue Cathode buffer, and the outer chamber was filled with anode buffer until it was 1/3 full. The gel was run at 4 °C for 60 min at 150 V. The dark blue cathode buffer was then replaced with the light blue cathode buffer, and the gel was run for an additional 90 min at 250 V. After electrophoresis was completed, the proteins were transferred to a membrane, as described in the “Immunoblotting” section.

For in-gel activity assays, an XCell SureLock Mini-Cell (Invitrogen) with a NativePAGE Novex 4-16% Bis–Tris gel (Invitrogen) was established, and 1× NativePAGE Anode buffer, NativePAGE light blue cathode buffer (1× NativePAGE running buffer, 0.1× cathode additive, 0.01% DDM, 0.05% sodium deoxycholate (DOC)) and NativePAGE cathode buffer (1× NativePAGE Running Buffer, 0.01% and DDM, 0.05% sodium deoxycholate (DOC)) were mixed. The wells of the gel were then filled with light blue cathode buffer, samples and NativeMark unstained protein standard (Invitrogen). The inner chamber was filled with light blue cathode buffer, and the outer chamber was filled with anode buffer until 1/3 full. The gel was run at 4 °C for 30 min at 100 V. The light blue cathode buffer was then replaced with cathode buffer, and the gel was run for an additional 4 h at 400 V. After electrophoresis was completed, the gel was treated with msolution (2.5 mg/mL nitrotetrazolium blue chloride (NTB), 2 mM Tris-HCl pH 7.4, and 0.1 mg/mL NADH) or Complex IV staining solution (0.5 mg/mL diaminobenzidine (DAB) and 1 mg/mL horse heart cytochrome c in 50 mM sodium phosphate, pH 7.2) until the bands were visualized (30 min to 24 h). After bands appeared, the reactions were stopped with 10% acetic acid, and the gels were imaged using a ChemiDoc MP Imaging System (Bio–Rad).

### Colocalization imaging of live cells

Cells were plated onto Nunc Lab-Tek 2-well chamber slides (Thermo Fisher Scientific) and allowed to adhere overnight. Plasmids encoding fluorescent-tagged proteins (dsRed-Mito, Addgene; *SIM2s-EGFP*, VectorBuilder; and *SIM2s-cherry*, VectorBuilder) were transiently transfected into cells using GeneJuice (EMD Millipore) according to the manufacturer’s protocol. Where applicable, MitoTracker Green FM (Invitrogen) was added to the cells at a concentration of 100 nM and incubated for 45 min at 37 °C prior to imaging. The cells were imaged on a Zeiss Stallion microscope with a ×63 plan apochromat objective. Line analysis was performed using ImageJ to generate signal intensity histograms.

### Proximity ligation assay

Protein interactions were analyzed by proximity ligation assay (PLA) using Sigma Duolink In Situ PLA technology following the manufacturer’s protocol. Briefly, pLPCX *SIM2s-FLAG* SUM159 cells were plated on glass coverslips and cultured to approximately 80% confluence before the experiment. To begin the experiment, cells were fixed in 2% p-formaldehyde (Santa Cruz) for 15 min and then permeabilized with 0.1% Triton X-100 in PBS for 15 min. The cells were blocked using Duolink Blocking Solution for 60 min at 37 °C and then incubated with primary antibody (see Supplementary Table 2 for antibody and dilution data) for 3 h at room temperature. After primary antibody incubation, the Duolink PLA probes anti-mouse PLUS (DUO92001) and anti-rabbit MINUS (DUO92005) were added to the cells and incubated at 37 °C for 1 h. Signals were then induced using Duolink In Situ Detection Reagents Orange (DUO92007) following the manufacturer’s ligation protocol for 30 min at 37 °C and then, for signal amplification, the protocol incubation was continued for 100 min at 37 °C. Following signal amplification, the cells were either stained with 16 mM Hoechst (Thermo Scientific) and mounted on glass microscope slides using Prolong Anti-fade Gold (Thermo Scientific), or the cover slips were mounted on glass microscope slides using Duolink In Situ Mounting Medium with DAPI. Images were taken with a Zeiss Axio Imager.Z1 with ×40 (quantification) and 63X (representative images) plan apochromat objectives.

### Coimmunoprecipitation

Cells were lysed in RIPA buffer supplemented with 1 mM Na_3_VO_4_, 1 mM cOmplete ULTRA Tablets, Mini, EDTA-free EASYPack (Roche), and 100 nM MG-132. Protein concentrations were measured using a DC Assay Kit (Bio–Rad), and 100 μg of protein was used for each sample. Samples were added to either mouse IgG magnetic beads (Cell Signaling) or to 6 μg of primary antibody and incubated at 4 °C overnight with gentle agitation. Samples containing antibody were added to cleared protein G beads (with active motifs) and incubated for 4 h at 4 °C with gentle agitation. All samples were then washed with TBS, and proteins were eluted from the beads by adding 2× nonreducing Laemmli buffer (250 mM Tris-HCl; 8% SDS; and 40% glycerol, pH 6.8) and incubation at 95 °C for 5 min. β-Mercaptoethanol was added to each sample, and immunoblotting was performed.

### MitoTracker live-cell imaging

Cells were plated onto Nunc Lab-Tek 2-well chamber slides (Thermo Fisher Scientific) and allowed to adhere overnight. MitoTracker Green FM (Invitrogen) was added to the cells at a concentration of 100 nM and incubated for 45 min at 37 °C. MitoTracker was then removed, and 20 μM Hoechst 33342 was added, and the culture were incubated for 5 min at 37 °C. Fresh phenol red-free medium was then added, and the cells were imaged with an Olympus FLUOVIEW FV3000 Confocal Laser Scanning Microscope using a ×60 plan apochromat objective. Images were analyzed using the MicroP program. Mitochondria identified as either small globe or large globe mitochondria via software analysis were considered to be fragmented, and all other mitochondrion types were considered to be fused.

### NAD^±^/NADH measurement

An NAD/NADH-Glo Assay (Promega) was performed to determine the NAD^+^/NADH ratio. Briefly, cells were dissociated from culture dishes with trypsin and resuspended in PBS at a concentration of 2 × 10^6^ cells per mL. Samples were split into 2 groups; in one-half of the samples, NAD^+^ was digested, and in one-half of the sample, NADH was digested. The reaction mixture was added, and luminescence was measured with a Synergy H1 (BioTek) microplate reader. The luminescence values of NAD^+^ and NADH in each sample were used to calculate NAD^+^/NADH ratio. A minimum of 4 samples were used for this analysis.

### MitoSOX assay

Superoxide production from mitochondria was measured using MitoSOX Red (Thermo Fisher Scientific) and quantified using a Synergy H1 microplate reader (BioTek). Cells were plated in 96-well plates and allowed to adhere overnight. Cells were then washed with PBS, and 5 μM MitoSOX reagent in phenol red-free medium was added and incubated for 10 min at 37 °C. Cells were rinsed with phenol red-free medium, and the fluorescence was measured (Ex 510/Em 580). Mean fluorescence values were calculated from a minimum of seven replicates.

### Statistical analysis

All experiments were performed in a minimum of biological triplicates. F tests were conducted to test for significant differences in the variance of each sample (significant at *P* < 0.05). When variances were not different, unpaired two-tailed Student’s *t* tests were performed, and when variances were different, unpaired two-tailed Welch’s *t* tests were performed. Western blot quantification was performed using paired two-tailed *t* tests. Normal distribution of the data was confirmed before conducting all tests, and differences between groups were considered to be significantly different when *P* < 0.05. All statistical tests were performed with the Prism (GraphPad) software.

## Results

**Fig. 1:**
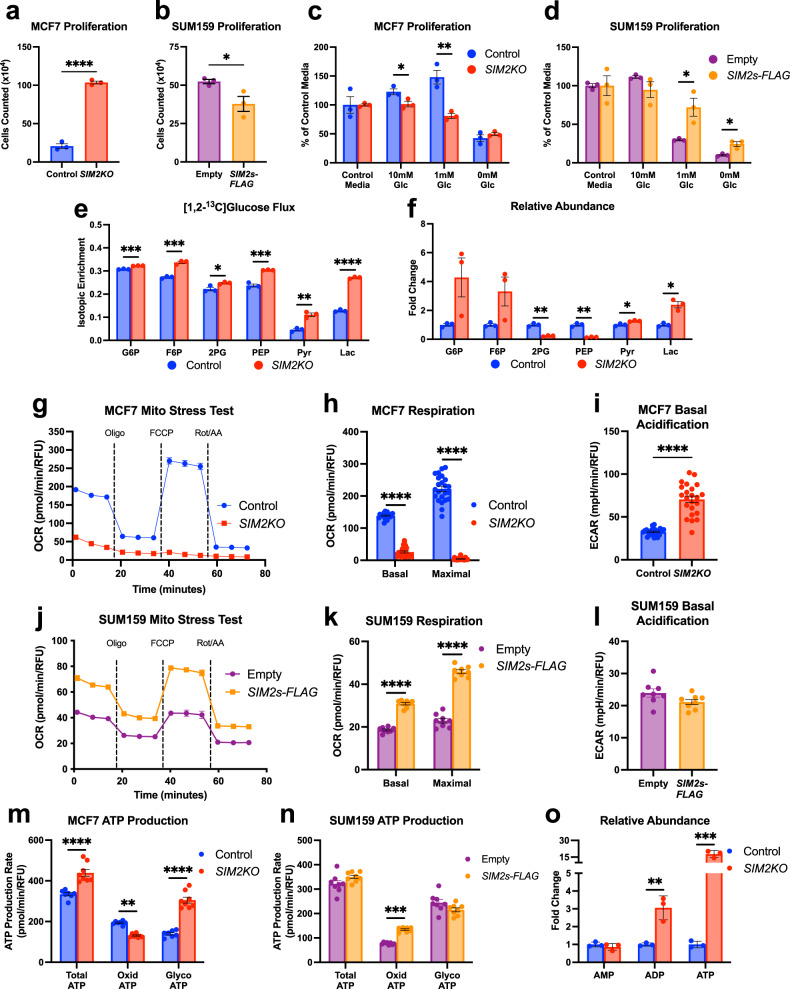
*SIM2* loss increases glycolytic metabolism in MCF7 cells, and *SIM2*s overexpression promotes oxidative phosphorylation. **a**–**d** Proliferation assays of **a** *SIM2KO* MCF7 and **b** *SIM2s-FLAG-*overexpressing SUM159 cells in normal medium. **c** *SIM2KO* MCF7 and **d** *SIM2s-FLAG* SUM159 cells were also grown in medium containing 10 mM, 1 mM, and 0 mM glucose, and growth is presented as the percentage of growth compared to that of cells in control medium. Cells were counted 3 days after plating, and comparisons were made to control cells under the given conditions. The data are presented as the means ± SEMs. *P < 0.05, **P < 0.01, ****P < 0.0001, two-tailed Student’s *t* test; *n* = 3 samples. **e** Stable isotopic tracing of [1,2-^13^C]-glucose is presented as isotopic enrichment of glucose-6-phosphate (G6P), fructose-6-phosphate (F6P), 2-phosphoglycerate (2PG), phosphoenolpyruvate (PEP), pyruvate (Pyr), and lactate (Lac) in *SIM2KO* MCF7 *cells*. The data are presented as the means ± SEMs. *P < 0.05, **P < 0.01, ****P < 0.0001, two-tailed Student’s *t* test; *n* = 3 samples. **f**, **o** Relative abundance of G6P, F6P, 2PG, PEP, Pyr, Lac, (**o**) AMP, ADP, and ATP as measured by the addition of the area under all peaks of each metabolite and normalized by the total peak intensity of each sample. The data are expressed as the expression fold change in *SIM2KO* MCF7 cells compared to MCF7 control cells ± SEM. **P < 0.01, ***P < 0.001, two-tailed Welch’s *t* test; *n* = 3 samples. **g**–**l** A mitochondrial stress test was performed using a Seahorse XF Ex*t*racellular Flux Analyzer on **g**–**i** *SIM2KO* MCF7 and **j**–**l** *SIM2s-FLAG* SUM159 cells. **g**, **j** Oxygen consumption rates (OCR) were plotted throughout the experiment. **h**, **k** The OCR after rotenone/antimycin-A treatment data were subtracted from the third measurement and the seventh measurement to obtain the basal and maximal respiration rates, respectively. **i**, **l** Basal extracellular acidification rates (ECAR) were measured. OCR and ECAR measurements are plotted as the mean ± SEM. ****P < 0.0001, two-tailed Student’s or Welch’s *t* test; *n* = 23 for MCF7 control cells, *n* = 22 for *SIM2KO* MCF7 cells, and *n* = 8 for all other cell samples. **m** For *SIM2KO* MCF7 cell and **n** *SIM2s-FLAG* SUM159 cells, the ATP production rates (*J*_ATP_) from glycolysis (*J*_ATPglyc_) and from oxidative phosphorylation (*J*_ATPox_) were determined using Wave software (Agilent) with input from the third and sixth OCR and ECAR measurements. J_ATP_ is plotted as the mean ± SEM. **P < 0.01, ***P < 0.001, ****P < 0.0001, two-tailed Student’s *t* test; *n* = 7 for MCF7 cell control samples and *n* = 8 for all other samples.

We have shown that loss of *SIM2s* in MCF7 and MCF10A cells and the mouse mammary gland induces an EMT^34–36,39,40^. Potential downstream targets were identified by microarray analysis of *shSIM2* MCF7 cells via ingenuity pathway analysis. Pathways critical to tumor metabolism and cellular function, as well as canonical pathways including p53 signaling, mitochondrial dysfunction, and oxidative stress, were altered with loss of *SIM2s* (Supplementary Fig. 1a). Additionally, the culture medium of *shSIM2* MCF7 cells showed acidification compared to that of the control cells one day after a similar number of cells were plated, suggesting that loss of *SIM2s* induces glycolysis (Supplementary Fig. 1b). To further determine the effect of *SIM2s* on metabolism, we generated CRISPR/Cas9 *SIM2* knock-out (*SIM2KO*) cells (Supplementary Fig. 1c), which also underwent an EMT (Supplementary Fig. 1d–g) and overexpressed FLAG-tag labeled *SIM2s* (*SIM2s-FLAG*) in SUM159 breast cancer cells that did not express *SIM2s*^43,44,48^. In normal glucose-containing medium, *SIM2KO* cells proliferated more rapidly than control cells (Fig. 1a and Supplementary Fig. 1h), whereas SIM2s inhibited the proliferation of SUM159 cells (Fig. 1b). When we inhibited glycolysis and forced the activation of oxidative phosphorylation (OXPHOS) via glucose starvation, we found that loss of *SIM2s* impaired cell adaptation to nutrient stress, whereas re-establishment of *SIM2s* expression promoted cell resistance to low glucose, in contrast to the effect in control cells (Fig. 1c, d and Supplementary Fig. 1i). This response is likely due to the ability of *SIM2s*-expressing cells to utilize mitochondria more efficiently than control or *SIM2KO* cells.

A hallmark of cancer described by Warburg^5^ is dysregulated energy metabolism in cancer cells, often indicated by an increased aerobic glycolysis rate and a decreased mitochondrial oxidative phosphorylation. To determine whether the *SIM2KO* cells underwent a metabolic shift, we measured the abundance of metabolites in the glycolytic pathway as well as the flux through this pathway using [1,2-^13^C] glucose tracing and ion chromatography with mass spectrometry (IC-MS) and found that *SIM2KO* cells showed increased glycolytic flux, as indicated by increased isotopic enrichment of glucose-6-phosphate (G6P), fructose-6-phosphate (F6P), 2-phosphoglycerate (2PG), phosphoenolpyruvate (PEP), pyruvate (Pyr), and lactate (Lac) (Fig. 1e). Furthermore, we observed that *SIM2KO* cells showed a decreased abundance in the glycolytic intermediates 2PG and PEP but an increase in the levels of the glycolysis products pyruvate and lactate, indicating that these cells underwent a metabolic shift to increase glycolysis, even under aerobic conditions (Fig. 1f). Considering these changes, we sought to determine whether SIM2s regulated the transcription of critical genes involved in glucose uptake and metabolism. Surprisingly, the mRNA expression of solute carrier family 2 member 1 (*SLC2A1*; GLUT1) and hexokinase 2 (*HK2*) was decreased in *SIM2KO* MCF7 cells compared to controls, but the expression of solute carrier family 2 member 4 (*SLC2A4*; GLUT4), lactate dehydrogenase A (*LDHA*) and lactate dehydrogenase B (*LDHB*) was elevated (Supplementary Fig. 2a–e). In *SIM2s-FLAG* SUM159 cells, *SLC2A1* mRNA expression was increased, *SLC2A4* and *HK2* expression was unchanged, and *LDHA* and *LDHB* expression decreased (Supplementary Fig. 2f–j). These results suggest that loss of *SIM2s* does not promote acquisition of a Warburg phenotype through transcriptional regulation.

To better understand the function of SIM2s in metabolism, we examined the oxygen consumption rate (OCR) and the extracellular acidification rate (ECAR) in *SIM2KO* MCF7 and *SIM2s-FLAG* SUM159 cells using a Seahorse Extracellular Flux Bioanalyzer as described^45^. Our data showed that loss of *SIM2s* in MCF7 cells significantly reduced basal and FCCP-stimulated maximal respiration compared to that in control cells (Fig. 1g, h and Supplementary Fig. 2k, l). Consistent with increased lactate levels produced during aerobic glycolysis, the basal ECAR was elevated in *SIM2KO* and *shSIM2* MCF7 cells (Fig. 1i and Supplementary Fig. 2m). In contrast, the OCR rates were elevated in *SIM2s-FLAG* SUM159 cells, leading to increased basal and maximal respiration compared to that of the control cells (Fig. 1j, k). The basal ECAR was unchanged after *SIM2s-FLAG* overexpression compared to that in the control cells (Fig. 1l). Using OCR and ECAR data, we measured adenosine triphosphate (ATP) production during glycolysis (*J*_ATPglyc_) and oxidative phosphorylation (*J*_ATPox_)^49^. We observed that in *SIM2KO* MCF7 cells, *J*_ATPglyc_ was substantially increased, leading to elevated total ATP production despite a decrease in *J*_ATPox_ (Fig. 1m). Overexpression of *SIM2s* in SUM159 cells increased *J*_ATPox_; however, *J*_ATPglyc_ and total ATP production remained unchanged (Fig. 1n). These findings suggest that loss of *SIM2s* leads to dysfunctional mitochondrial respiration, resulting in an increase in aerobic glycolysis and glycolytic ATP production, whereas increased *SIM2s* expression promotes ATP production mediated through an increased OXPHOS rate. Moreover, an IC-MS analysis of relative adenosine monophosphate (AMP), adenosine diphosphate (ADP), and ATP abundance showed that+ ADP and ATP levels were increased in *SIM2KO* MCF7 cells (Fig. 1o). Taken together, our findings suggest that SIM2s drives OXPHOS and that loss of *SIM2s* expression shifts energy metabolism toward aerobic glycolysis, leading to increased cell proliferation.

### SIM2s does not alter OXPHOS through the regulation of mitochondrial protein abundance

PPARG coactivator 1 alpha (*PPARGC1A*) is a key transcriptional regulator of metabolism and mitochondrial biogenesis. We therefore examined the effect of SIM2s on the expression of PPARGC*1A* and found that it was elevated in *SIM2KO* MCF7 cells and unchanged in *SIM2s-FLAG* SUM159 cells (Supplementary Fig. 3a, b). Moreover, we found that SIM2s did not impact mitochondrial content, as indicated by immunoblot assay for measuring translocase of outer membrane subunit 70 (TOM70), which is located on the outer mitochondrial membrane (Supplementary Fig. 3c). We then hypothesized that SIM2s may alter OXPHOS by regulating the expression of mitochondrial respiratory chain (MRC) complexes and/or ATP synthase, which is also called Complex V (CV). MRC comprises four complexes: NADH:ubiquinone oxidoreductase (Complex I; CI), succinate dehydrogenase (Complex II; CII), ubiquinol–cytochrome c oxidoreductase (Complex III; CIII), and cytochrome c oxidase (Complex IV; CIV). An immunoblot analysis of MRC complex subunits in the extracts of whole *SIM2KO* MCF7 cells showed a reduction in Complex IV and I subunit levels and increased Complex II subunit expression (Supplementary Fig. 3c). The expression of MRC complex subunits was unchanged in *SIM2s-FLAG* SUM159 cells compared to that in control cells (Supplementary Fig. 3d). Although a reduction in MT-CO2 and NDUFB8 levels was observed with the loss of *SIM2s*, the overexpression of *SIM2s* did not enhance the protein expression of the ETC complex subunits evaluated. We therefore hypothesized that SIM2s affects the assembly and structure of MRCs not the expression of MRC proteins.

### SIM2s enhances MRC activity through the regulation of complex and supercomplex formation

To determine whether SIM2s affects MRC complex assembly, we performed blue native polyacrylamide gel electrophoresis (BN-PAGE) with digitonin-solubilized mitochondrial extracts (1:8 protein to digitonin) obtained from *SIM2KO* and *SIM2s-FLAG* MCF7 cells and immunoblot analysis with a cocktail of antibodies against OXPHOS-related proteins ATP5A (CV), MT-CO2 (CIV), UQCRC2 (CIII), SDHB (CII), and NDUFB8 (CI). This “shotgun” method to determine overall MRC formation changes revealed that SIM2s affected the levels of CI, CIII, and CIV and supercomplexes containing these complexes. Loss of *SIM2s* altered the composition and abundance of Complex I (CI)-containing supercomplexes (CI SCs) and reduced the formation of CIII and CIV (Supplementary Fig. 3e). In contrast, *SIM2s* overexpression led to an increase in the number of CI SCs as well as CIII and CIV abundance (Supplementary Fig. 3f). Examination of the MRC subunit proteins by SDS‒PAGE and immunoblotting revealed a similar expression pattern to that of extracts for both whole *SIM2KO* MCF7 and *SIM2s-FLAG* SUM159 cells (Supplementary Fig. 3g, h).

**Fig. 2:**
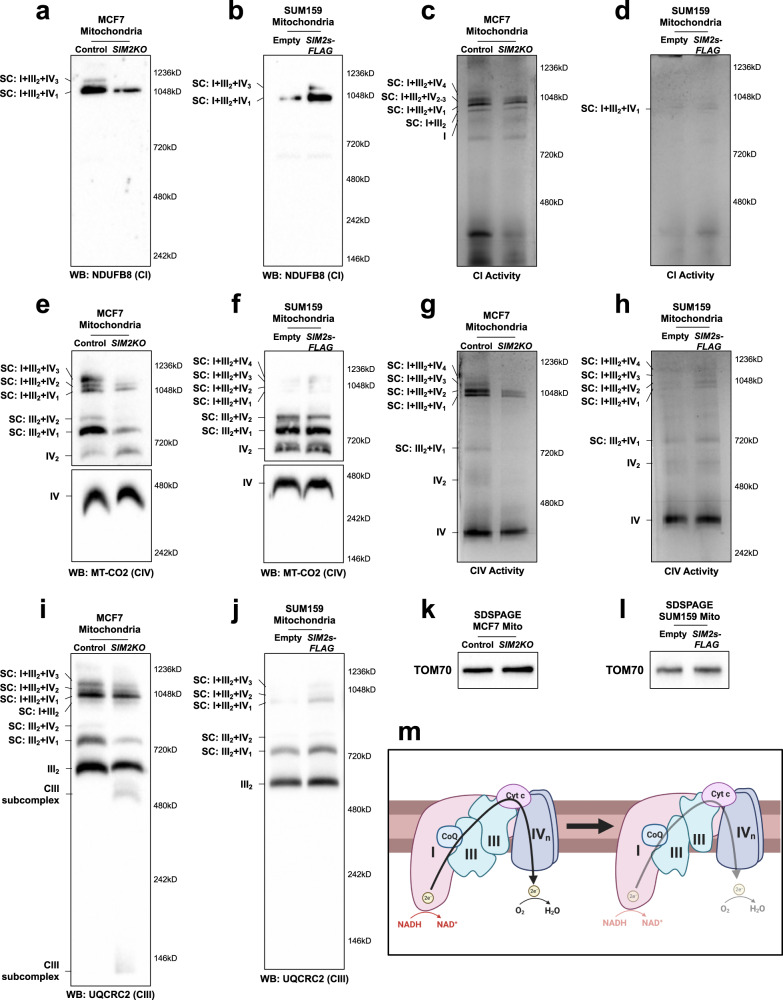
*SIM2* expression levels alter MRC complex and supercomplex (SC) formation and activity. Mitochondrial proteins shown in **a**, **e**, **i** are from *SIM2KO* MCF7 cells, and and those shown in **b**, **f**, **j** are from *SIM2s-FLAG* SUM159 cells. All cells were solubilized with 1:8 protein:digitonin, and the proteins were subjected to BN-PAGE followed by immunoblot analysis to measure the levels of **a**, **b** NDUFB8, **e**, **f** MT-CO2, and **i**, **j** UQCRC2. Mitochondrial proteins from **c, g** *SIM2KO* MCF7 cells and **d**, **h** *SIM2s-FLAG* SUM159 cells were solubilized with 1:8 protein:digitonin and subjected to CN-PAGE followed by an in-gel activity assay of **c**, **d** Complex I visualized by NTB oxidation and **g**, **h** Complex IV visualized by DAB oxidation. **k**, **l** SDS‒PAGE was followed by immunoblotting for measuring the level of the mitochondrial protein TOM70 in **k** *SIM2KO* MCF7 cells and **l** *SIM2s-FLAG* SUM159 cells. **m** Malformation of CIII inhibits electron flow through the MRC.

To explore the effect of SIM2s expression on MRC formation, we examined CI SC formation by BN-PAGE analysis followed by immunoblot analysis of NDUFB8 level. NDUFB8 incorporation into SC I + III_2_ + IV_1_ and SC I + III_2_ + IV_3_ was reduced in *SIM2KO* MCF7 cell mitochondria and increased in *SIM2s-FLAG* SUM159 cell mitochondria compared to that in control cells (Fig. 2a, b). Interestingly, clear native polyacrylamide electrophoresis (CN-PAGE) followed by an in-gel activity (IGA) assay of Complex I in MCF7 cell mitochondria revealed that *SIM2s* loss did not affect the ability of CI to oxidize NADH (Fig. 2c). An IGA revealed that CI activity in SUM159 cell mitochondria was low, and *SIM2s* overexpression did not change CI activity in the only observable supercomplex, SC I + III_2_ + IV_1_ (Fig. 2d). We next examined Complex IV by measuring MT-CO2 by immunoblotting after performing BN-PAGE and observed that an unassociated monomeric form of CIV was the most abundant form in the mitochondria of MCF7 and SUM159 cells, and *SIM2s* expression did not alter its level (Fig. 2e, f). Since monomeric CIV was highly prevalent, longer exposure of the membranes carrying proteins greater than 480 kDa was required to visualize CIV dimers and SCs (Fig. 2e, f). An analysis of CIV-containing species showed that the number of CIV dimers were increased in *SIM2KO* mitochondria compared to that in control mitochondria, while MT-CO2 incorporation into all the SC species assessed was reduced (Fig. 2e). *SIM2s* overexpression did not affect MT-CO2 incorporation into CIV dimers or SCIII_2_ + IV_1_ and III_2_ + IV_2_ but did increase MT-CO2 incorporation into CI SCs (Fig. 2f). Complex IV IGA assays with *SIM2KO* MCF7 cell mitochondria showed a dramatic loss of overall CIV activity, in CIV dimer activity and SC activity (Fig. 2g). An IGA of *SIM2s-FLAG* SUM159 cell mitochondrial CIV showed increased CIV activity in CIV monomers, CIV dimers, and all CIV-containing SC species (Fig. 2h). These data suggest that SIM2s supports CI-containing SC stabilization and CIV incorporation into SCs. In contrast, the capacity to transport electrons to CI through CIV correlated with SIM2 expression, particularly in SCs.

BN-PAGE followed by immunoblotting for measuring the Complex III subunit UQCRC2 revealed that *SIM2s* loss in MCF7 cells resulted in a minimal decrease in CIII incorporation in CI-containing SCs but a marked reduction in CIII incorporation in SCIII_2_ + IV_1_ and III_2_ + IV_2_ compared to controls (Fig. 2i). Moreover, subcomplexes of CIII were apparent in *SIM2KO* MCF7 cell samples, indicating incomplete formation of CIII (Fig. 2i). In contrast, no CIII subcomplexes were observed in SUM159 cell mitochondria, and the abundance of CIII-containing SCs was increased in *SIM2s*-overexpressing cells than in control cells (Fig. 2j). Importantly, the mitochondrial samples used for BN-PAGE and CN-PAGE analyses in this figure contained equal amounts of mitochondria, as indicated by the immunoblots for detecting TOM70 after SDS‒PAGE (Fig. 2k, l). Our results reveal that SIM2s promotes the proper assembly of CIII and SCs, and therefore, it appears to be critical for electron transfer in SCs (Fig. 2m).

### Mitochondrial localization of SIM2s leads to stabilized SCs

**Fig. 3:**
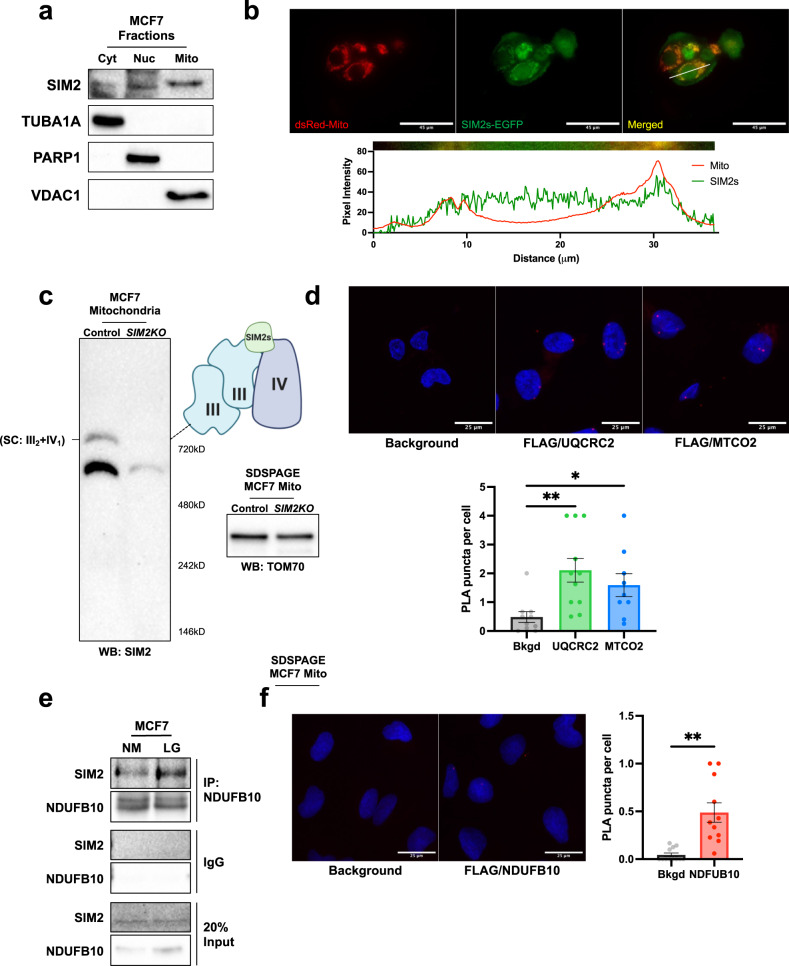
SIM2s localizes to mitochondria to facilitate SC formation through interactions with CI, CIII, and CIV. **a** MCF7 cells were fractioned into cytosol (Cyt), nuclear (Nuc), and mitochondrial (Mito) fractions via differential centrifugation. Immunoblot analysis for measuring SIM2 shows that it was expressed in all three cell fractions. Antibodies against TUBA1A, PARP and VDAC1 were used to determine the purity of the cytosolic, nuclear, and mitochondrial fractions, respectively. **b** MCF7 cells were cotransfected with *dsRed-Mito* and *EGFP-SIM2s* plasmids and imaged 24 h later. Histograms of each channel were generated using Fiji, and a line analysis was plotted. **c** *SIM2KO* MCF7 cell mitochondrial proteins were solubilized with 1:8 digitonin:protein and analyzed using BN-PAGE followed by immunoblotting for measuring the SIM2 level. Graphics depict the interaction of SIM2s and SC. SDS‒PAGE followed by immunoblotting for measuring TOM70 confirms equal mitochondrial protein content. **d** PLA with *SIM2s-FLAG* SUM159 cells using anti-FLAG and anti-UQCRC2 or anti-MT-CO2 antibodies. Positive interactions are indicated by red puncta, and nuclei are stained with Hoechst and imaged at 60x magnification with a confocal microscope. Positive puncta per cell are plotted showing the means ± SEMs. *P < 0.05, **P < 0.01, two-tailed Welch’s *t* test; *n* = 10 background images, *n* = 11 FLAG/UQCRC2 images, and *n* = 9 FLAG/MT-CO2 images. **e** MCF7 cells were grown in regular growth medium (NM) or 0.5 mM glucose medium (LG) for 24 h before coimmunoprecipitation (IP) using an anti-NDUFB10 antibody. Protein from IP samples, the IgG control, and input controls were analyzed by immunoblotting using anti-SIM2 and anti-NDUFB10 antibodies. **f** Proximity ligation assay (PLA) with *SIM2s-FLAG* SUM159 cells using anti-FLAG and anti-NDUFB10 antibodies. Positive interactions are indicated by red puncta, and nuclei are stained with Hoechst and imaged at 60x magnification with a confocal microscope. Positive puncta per cell are plotted showing the means ± SEMs. **P < 0.01, two-tailed Welch’s *t* test; *n* = 12 background images, and *n* = 11 FLAG/NDUFB10 images.

We have shown that SIM2s is required for mitochondrial function and MRC-containing SC formation. Noncanonical functions of nuclear transcription factors, including P53, STAT3, STAT5, NF-kB, HIF1A and steroid receptors, in mitochondria have been shown to regulate numerous mitochondrial processes independent of their nuclear activity^50–54^. We have previously shown that SIM2s exhibits nontranscriptional regulatory functions through protein‒protein interactions^44^. Moreover, SIM2 staining in human ductal carcinoma in situ (DCIS) lesions revealed that SIM2 expression was not exclusive to the nucleus^38,44^. To determine whether SIM2s physically localizes to the mitochondria, we fractionated wild-type MCF7 cells by differential centrifugation and examined cytosolic (Cyt), nuclear (Nuc), and mitochondrial (Mito) fractions for SIM2s (Fig. 3a). As expected, we found that SIM2s was highly expressed in the nuclear fraction and was present in the mitochondrial fraction. Tubulin alpha 1A chain (TUBA1A), poly [ADP-ribose] polymerase 1 (PARP1), and voltage-dependent anion-selective channel protein 1 (VDAC1) were clearly expressed in only the cytosolic, nuclear, and mitochondrial fractions, respectively (Fig. 3a). To confirm the SIM2s mitochondrial localization, we performed coimmunofluorescence with MCF7 cells cotransfected with dsRed-Mito and *SIM2s*-EGFP plasmids. The mitochondrial marker dsRed-Mito appeared as distinct puncta in mitochondria, and SIM2s-EGFP fluorescence was observed in the nucleus as well as mitochondria, as indicated by the yellow signal in the merged image (Fig. 3b). Moreover, a line analysis confirmed that SIM2s and mitochondrial peaks overlapped, indicating colocalization (Fig. 3b).

To better understand the mechanism by which SIM2s promotes mitochondrial MRC-containing SC formation, we ran BN-PAGE followed by Western blotting for measuring SIM2s in MCF7 cell control and *SIM2KO* mitochondria. SIM2s bands were observed with the same molecular weight as a SC III_2_ + IV_1_, suggesting that SIM2s is part of the SC assembly (Fig. 3c). To determine whether SIM2s interacts with CIII or CIV, we performed a proximity ligation assay (PLA) with *SIM2s-FLAG* SUM159 cells. The presence of red puncta indicated that SIM2s interacts with the CIII (UQCRC2) and CIV (MT-CO2) subunits (Fig. 3d).

Examining mass spectrometry data of SIM2s-overexpressing SUM159 cells, we found that SIM2s was associated with a number of mitochondrial proteins (Supplementary Table 1); one of these proteins was NDUFB10 (NADH dehydrogenase [ubiquinone] 1 beta subcomplex subunit 10), which is a peripheral membrane subunit of CI and is critical to CI formation^30^. We immunoprecipitated NDUFB10 using protein lysates from MCF7 cells, which had been treated with control and low-glucose medium, and blotted the proteins pulled down with SIM2s. We found that SIM2s was present in the NDUFB10-immunoprecipitated samples, with stronger interactions obtained under low-glucose conditions (Fig. 3e). Additionally, we confirmed the SIM2s-NDUFB10 interaction in *SIM2s-FLAG* SUM159 cells by PLA. We observed red puncta indicating SIM2s-NDUFB10 interactions (Fig. 3f). Considering these data, we demonstrate that SIM2s directly interacts with CI, CIII, and CIV subunits, likely facilitating functional SC assembly.

### SIM2s interaction is required for MRC CIII stability

**Fig. 4:**
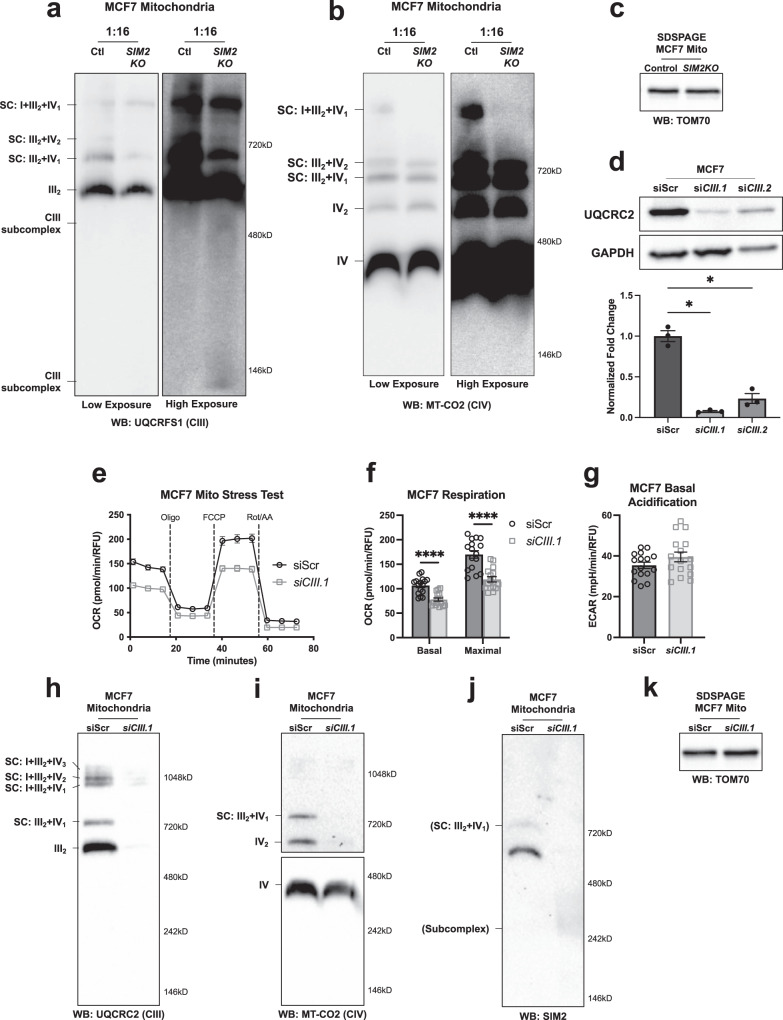
SIM2s enhances CIII stability and incorporation into supercomplexes. **a**, **b** Mitochondrial proteins from *SIM2KO* MCF7 cells were solubilized with 1:16 protein: digitonin and subjected to BN-PAGE followed by immunoblot analysis to measure the level of **a** UQCRFS1 and **b** MT-CO2. Low and high exposure are used to display all MRC species. **c** SDS‒PAGE followed by immunoblotting for measuring the TOM70 mitochondrial protein level in *SIM2KO* MCF7 cells. **d** Immunoblot showing UQCRC2 in MCF7 cells transfected with siRNAs targeting *UQCRC2* (*siCIII.1* and *siCIII.2*). Densitometry analysis was performed using Fiji, and the fold change in UQCRC2 level was normalized to the level of GAPDH and is plotted as the mean ± SEM. *P < 0.05, paired two-tailed *t* test; *n* = *3*. **e-g** A mitochondrial stress test was performed using a Seahorse XF Extracellular Flux Analyzer with MCF7 cells transfected with *siCIII.1*. **e** Oxygen consumption rates (OCR) were plotted throughout the experiment. **f** OCR after rotenone/antimycin-A treatment data were subtracted from the third measurement and seventh measurement to obtain the basal and maximal respiration rates, respectively. **g** Basal extracellular acidification rates (ECARs) were measured. OCR and ECAR measurements are plotted as the mean ± SEM. ****P < 0.0001, two-tailed Student’s or Welch’s *t* test; *n* = 16. **h**–**j** Mitochondrial proteins from MCF7 cells transfected with *siCIII.1* were solubilized with 1:8 protein:digitonin and subjected to BN-PAGE followed by immunoblot analysis of the level of **h** UQCRFS1, **i** MT-CO2, and **j** SIM2. **k** SDS‒PAGE followed by immunoblotting for measuring the TOM70 mitochondrial protein level shown in **h**–**k**.

Our data reveal that SIM2s loss alters CIII formation, leading to the malformation of supercomplexes (SCs) (Fig. 2). A BN-PAGE analysis of CIII and subsequent immunoblotting to measure the CIII subunit, UQCRFS1, level was performed with *SIM2KO* MCF7 cell mitochondria solubilized with an increased protein:digitonin ratio (1:16) compared to that used in previous analysis (Fig. 2i). Loss of *SIM2s* reduced the formation of the CIII dimer, SC CIII_2_ + IV_1_ and SC CIII_2_ + IV_2_ and increased the levels of CIII assembly intermediates (Fig. 4a). Examination of CIV levels after 1:16 protein digitonin mitochondrial solubilization confirmed that no CIV subassemblies were formed in the control or *SIM2KO* MCF7 cells (Fig. 4b). SDS‒PAGE followed by immunoblotting for measuring TOM70 levels showed equal mitochondrial concentrations in the samples used that are presented in Fig. 4a, b (Fig. 4c). These data further showed that SIM2s promotes CIII and SC assembly. We then employed *UQCRC2*-targeting siRNAs to knock down CIII in MCF7 cells and demonstrate the necessity of UQCRC2 for CIII and SC formation. siRNA UQCRC2 #1 (*siCIII.1*) was the most effective siRNA for reducing the expression of the CIII and SC proteins and was therefore used in subsequent experiments (Fig. 4d). Seahorse analysis demonstrated that *siCIII.1* reduced basal and maximal OCR without changing ECAR compared to these rates in the control MCF7 cells, as expected (Fig. 4e–g). BN-PAGE followed by immunoblotting for UQCRC2 in MCF7 *siCIII.1* mitochondria confirmed that UQCRC2 was markedly reduced, with only faint bands appearing for the CIII dimer and CI-containing SCs (Fig. 4h). BN-PAGE followed by immunoblotting for MT-CO2 in MCF7 *siCIII.1* mitochondria revealed reduced formation of CIV dimers and SC CIII_2_ + IV_1_ but not individual CIV monomers (Fig. 4i). Similarly, immunoblotting for SIM2s levels in MCF7 *siCIII.1* mitochondria after BN-PAGE showed that SIM2s incorporation into SC CIII_2_ + IV_1_ was abrogated and that SIM2s was found in MRC subassemblies (Fig. 4j). SDS‒PAGE followed by immunoblotting for measuring TOM70 levels in MCF7 siScr and *siCIII.1* mitochondria confirmed the equal mitochondrial content in the samples used for the BN-PAGE analysis (Fig. 4k). Our results show that the interaction between SIM2s and UQCRC2 is critical for the formation and activity of SC CIII_2_ + IV_1_.

### Loss of *SIM2s* mediates SC dysfunction and alters mitochondrial networks

**Fig. 5:**
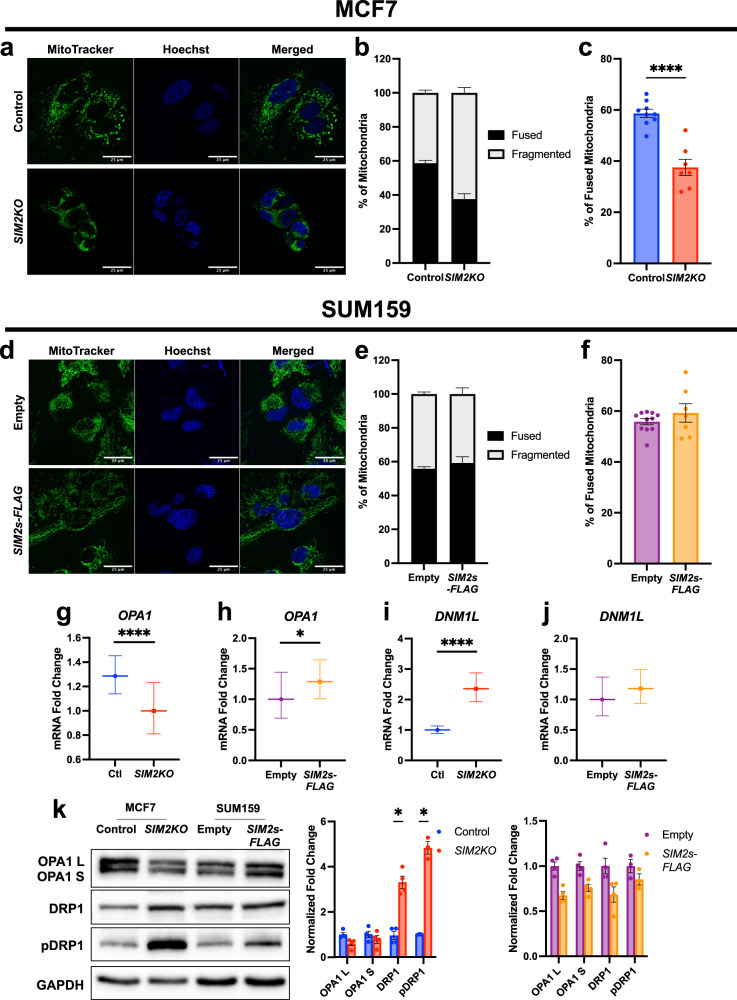
Loss of *SIM2s* promotes mitochondrial fragmentation. Confocal images showing **a** *SIM2KO* MCF7 and **d** *SIM2s-FLAG* SUM159 cells stained with MitoTracker Green FM to observe mitochondrial structure and with Hoechst to identify cell nuclei. **b**, **c**, **e**, **f** MitoTracker images were the inputs for MicroP software analysis, which was performed to unbiasedly count fused and fragmented mitochondria. **b**, **e** Distribution of fused and fragmented mitochondria is displayed in a bar chart. **c**, **f** The percentage of fused mitochondria per image is plotted to show the mean ± SEM. ****P < 0.0001, two-tailed Student’s *t* test; *n* = 9 control cell images, *n* = 7 *SIM2KO*, *n* = 11 empty cell images, and *n* = 7 *SIM2s-FLAG* images. **g**–**j** Quantitative RT–PCR analysis of **g**, **h** *OPA1* and **i**, **j** *DNM1L* level in *SIM2KO* MCF7 and *SIM2s-FLAG* SUM159 cells. The data were analyzed by the 2^−ΔΔCt^ method, and the error is presented as the transformed sum of the squares of the SD of the target and reference genes. *P < 0.01, two-tailed Student’s *t* test of ΔCt values; *n* = 3 samples. **k** Immunoblot analysis showing *SIM2KO* MCF7 and *SIM2s-FLAG* SUM159 cell proteins using antibodies against OPA1, DRP1, pDRP1(S673), and GAPDH. Densitometry analysis was performed using Fiji, and the fold change in each protein level was normalized to that of GAPDH and is plotted showing the mean ± SEM. *P < 0.05, paired two-tailed *t* test; *n* ≥ *3*.

The mitochondrial network in cells is maintained through constant fusion and fission mediated by mitochondrial dynamin-like GTPase (OPA1) and dynamin-1-like protein (DRP1), respectively. Mitochondrial fusion has been shown to be associated with increases in mitochondrial respiration and mitochondrial membrane potential, while mitochondrial fission (or fragmentation) has been shown to be associated with decreased OXPHOS rates and to be a major mechanism in which damaged portions of the mitochondrial network are sequestered and degraded through mitophagy^32,55–57^. To determine whether mitochondrial dynamics are impacted by SIM2s, we analyzed *SIM2KO* MCF7 and *SIM2s-FLAG* SUM159 cells stained with MitoTracker Green. The results showed that loss of *SIM2s* led to fragmentation of the mitochondrial network and a reduction in the number of elongated mitochondria (Fig. 5a–c). Interestingly, overexpression of *SIM2s-FLAG* did not alter the mitochondrial network in SUM159 cells (Fig. 5d–f). We then measured the mRNA expression of *OPA1* and *DNM1L* (gene encoding DRP1) in *SIM2KO* MCF7 and *SIM2s-FLAG* SUM159 cells and observed that *OPA1* was reduced with the loss of *SIM2s* and was elevated with overexpression of *SIM2s-FLAG* (Fig. 5g, h). *DNM1L* expression was increased in our *SIM2KO* MCF7 cells and was unchanged in the *SIM2s-FLAG* SUM159 cells (Fig. 5i, j). An immunoblot analysis of *SIM2KO* MCF7 and *SIM2s-FLAG* SUM159 cell proteins showed that OPA1 expression was unaffected by *SIM2s* expression (Fig. 5k). Total DRP1 protein was elevated in *SIM2KO* MCF7 cells and unchanged in *SIM2s-FLAG* SUM159 cells (Fig. 5k). Interestingly, phosphorylation of DRP1 at serine 637 has been shown to reduce mitochondrial fission^58^; however, Tomkova et al. found that phosphorylation of DRP1 at S637 was associated with fragmented mitochondria in MCF7 and T47D cells with SC assembly and OXPHOS defects^32^. Similarly, we observed that pDRP(S637) expression was elevated in *SIM2KO* MCF7 cells, which also acquired a fragmented mitochondrial phenotype, and was unchanged in *SIM2s-FLAG* SUM159 cells (Fig. 5k). These results suggest that phosphorylation of DRP1 at serine 637 promotes DRP1 activity, not inhibition, in response to the loss of OXPHOS associated with SC disassembly. Moreover, similar to our observations with *SIM2KO* MCF7 cells, dual upregulation of DRP1 and PGC1α has been previously shown in breast cancer cells and has been associated with a reduced OXPHOS rate and increased mitochondrial turnover^59^.

### Flux through the TCA cycle is altered with loss of *SIM2s* due to changes in NADH utilization

**Fig. 6:**
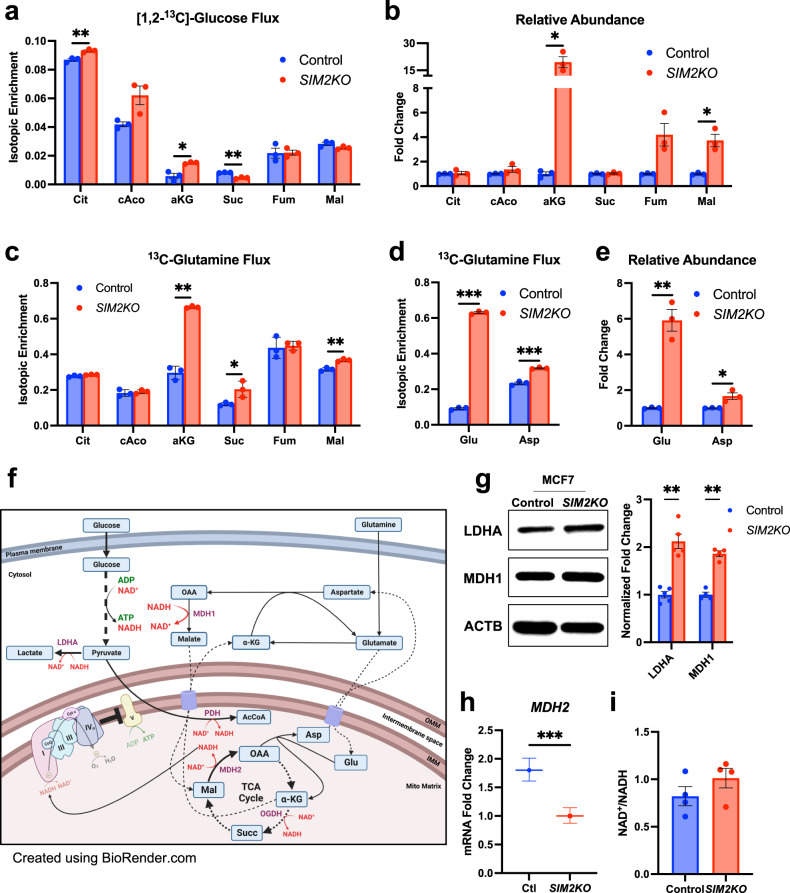
*SIM2* loss alters metabolic flux in mitochondria. Stable isotopic tracing of **a** [1,2-^13^C2]-glucose presented as the isotopic enrichment of citrate (Cit), *cis*-aconitase (cAco), alpha-ketoglutarate (aKG), succinate (Suc), fumarate (Fum), and malate (Mal) in *SIM2KO* MCF7 cells. Data are presented as the means ± SEMs. *P < 0.05, **P < 0.01, Student’s *t* test; *n* = 3 samples. **b**, **e** Relative abundance of **b** Cit, cAco, aKG, Suc, Fum, Mal, glutamate (Glu), and aspartate (Asp) as measured by addition of the area under all peaks of each metabolite and normalized to the total peak intensity of each sample. Data are expressed as *SIM2KO* MCF7 cell protein expression fold change compared to the protein expression in the control cells ± SEM. *P < 0.05, **P < 0.01, ***P < 0.001, two-tailed Welch’s *t* test; *n* = 3 samples. **c**, **d** Stable isotopic tracing of [^13^C5]-glutamine presented as the isotopic enrichment of **c** Cit, cAco, aKG, Suc, Fum, Mal, **d** Glu, and Asp in *SIM2KO* MCF7 cells. Data are presente**d** as the means ± SEMs. *P < 0.05, **P < 0.01, ***P < 0.001, two-tailed Welch’s *t* test for aKG and Student’s *t* test for all others; *n* = 3 samples. **f** Schematic showing the relevant metabolic reactions and pathways using NAD^+^ and NADH and how they are altered by the loss of *SIM2s*. **g** *SIM2KO* MCF7 cell protein was analyzed by immunoblotting using antibodies against LDHA, MDH1, and ACTB. Densitometry analysis was performed using Fiji, and the fold changes in LDHA and MDH1 levels were normalized to the level of ACTB and are plotted as the means ± SEMs. **P < 0.01, paired two-tailed *t* test; *n* = 4. **h** Quantitative RT‒PCR analysis of *SIM2KO* MCF7 cells showing relative mRNA expression of *MDH2*. The data were analyzed by the 2^-ΔΔCt^ method, and the error is presented as the transformed sum of the squares of the SD of the target and reference genes. ***P < 0.001, two-tailed Student’s *t* test of ΔCt values; *n* = 3 samples. **i** NAD^+^/NADH ratio calculated using NAD^+^ and NADH concentrations measured from the same samples with a NAD/NADH-glo assay kit. Data are presented as the mean ratio ± SEM. *P* = 0.2325 Two-tailed Student’s *t* test; *n* = 4 samples.

The canonical metabolic pathway connecting OXPHOS and glycolysis is the tricarboxylic acid (TCA) cycle. Pyruvate from glycolysis can enter mitochondria and contribute to either the TCA cycle through conversion to acetyl-CoA by the pyruvate dehydrogenase complex (PDH) or can be converted to lactate. We showed that pyruvate and lactate production via glycolysis was elevated with the loss of *SIM2s* (Fig. 1e, f). We then examined the contribution of glucose to the TCA cycle in *SIM2KO* MCF7 cells by [1,2-^13^C]-glucose tracing and IC-MS analysis of targeted TCA cycle intermediates (Fig. 6a). Surprisingly, isotopic enrichment of citrate (Cit) and alpha-ketoglutarate (aKG) was elevated, while that of succinate (Suc) was decreased in the *SIM2KO* cells, suggesting that the TCA cycle was at least partially active after the loss of MRC activity (Fig. 6a). Interestingly, the abundance of total aKG was markedly elevated, along with that of malate, while the levels of all other measured TCA metabolites were unchanged in the *SIM2KO* cells (Fig. 6b). The marked increase in aKG level indicates that the majority of aKG is not synthesized from glucose but from a different carbon source. Indeed^13^,C-glutamine tracing and IC-MS revealed isotopic enrichment of aKG, succinate, and malate in *SIM2KO* MCF7 cells, while the levels of other TCA metabolites remained unchanged (Fig. 6c). Moreover^13^,C-glutamine tracing of glutamate (Glu) and aspartate (Asp) revealed significant increases in both isotopic enrichment and abundance upon loss of *SIM2s* (Fig. 6d, e). Elevated levels of glutamine-derived and overall glutamate, aKG, aspartate and malate suggest increased glutaminolysis and malate–aspartate shuttle (MAS) activity^11,60,61^. The MAS transports NAD(H) across the inner mitochondrial membrane to supply NADH to Complex I and NAD^+^ to glycolytic enzymes^62^. Given that loss of *SIM2s* disrupts MRC formation and inhibits OXPHOS, we postulate that increased NADH metabolism via the MAS is required for compensatory ATP production via glycolysis (Fig. 6f). We observed a shift in MAS activity toward cytosolic production of NAD^+^, as indicated by the increased protein expression of the cytosolic enzymes LDHA and MDH1 and reduced gene expression of NADH-producing mitochondrial malate dehydrogenase (*MDH2*) in *SIM2KO* MCF7 cells (Fig. 6g, h). However, we observed no change in NAD^+^/NADH between control and *SIM2KO* cells (Fig. 6i). Our results show that cells with impaired OXPHOS due to the disruption of SC formation respond by modulating NADH metabolism via glycolysis and glutaminolysis.

### Glutaminolysis provides building blocks for the rapid proliferation of *SIM2KO* cells

**Fig. 7:**
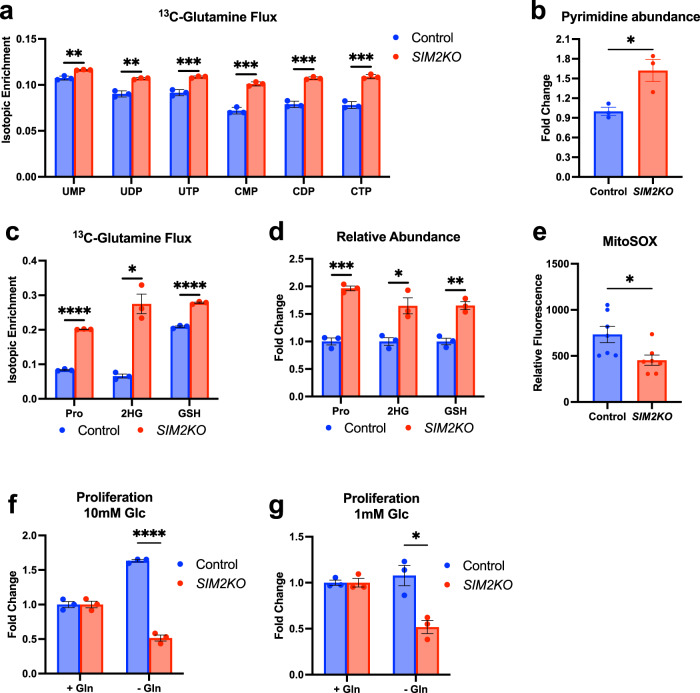
Increased formation of cell proliferation-promoting metabolites derived from glutamine in *SIM2KO* cells. **a**, **c** Stable isotopic tracing of [^13^C5]-glutamine presented as the isotopic enrichment of **a** UMP, UDP, UTP, CMP, CDP, CTP, **c** proline (Pro), 2-hydroxyglutarate (2HG), and glutathione (GSH) in *SIM2KO* MCF7 cells. Data are presented as the means ± SEMs. **P < 0.01, ***P < 0.001, ****P < 0.0001, two-tailed Student’s *t* test; *n* = 3 samples. Relative abundance of **b** all pyrimidine-containing meta**b**olites (UMP, UDP, UTP, CMP, CDP, CTP), **d** Pro, 2HG, and GSH measured by addition of the area under all peaks of each metabolite and normalized to the total peak intensity of each sample. Data are expressed as *SIM2KO* MCF7 cell protein expression fold change compared to the protein expression level of the control cells ± SEM. *P < 0.05, **P < 0.01, ***P < 0.001, two-tailed Student’s *t* test; *n* = 3 samples. **e** Fluorescence signal from MitoSOX accumulation measured at 510 excitation and 580 emission. Data are plotted as the mean relative fluorescence ± SEM. *P < 0.05, two-tailed Student’s *t* test; *n* = 7 samples. **f**, **g** SIM2KO MCF7 cells were grown in 10 mM glucose-supplemented or **b** 1 mM glucose-supplemented medium with or without 2 mM glutamine. Cell counts were normalized to the number in glutamine-containing medium for each cell line and are represented as the number fold changes ± SEM. *P < 0.05, ****P < 0.0001, two-tailed Student’s *t* test; *n* = 3 samples.

In addition to the role played by glutamine in promoting glycolysis through NAD(H) generation, other roles of glutaminolysis include provision of amino acids for protein synthesis and pyrimidines for nucleic acid synthesis^9,11^. The first step of glutaminolysis is the conversion of glutamine to glutamate, which, as previously shown, increases the level of ^13^C-glutamine and overall glutamine abundance in *SIM2KO* cells (Fig. 6d, e). Additionally, we observed that the level of aspartate, which plays a role in the generation of various amino acids as well as pyrimidines^63^, was elevated via glutaminolysis in *SIM2KO* MCF7 cells (Fig. 6d, e). All the pyrimidine-containing compounds derived from^13^C-glutamine, including UMP, UDP, UTP, CMP, CDP, and CTP, showed increased isotopic enrichment in *SIM2KO* cells (Fig. 7a). The overall pyrimidine abundance was also elevated, suggesting that pyrimidine synthesis through glutaminolysis was increased, allowing increased proliferation in *SIM2KO* cells (Fig. 7b). We also measured ^13^C-glutamine incorporation into proline and 2-hydroxyglutarate (2HG), which are two oncogenic metabolites in breast cancer^64–66^. In *SIM2KO* MCF7 cells we observed increased isotopic enrichment and abundance of both proline and 2HG in *SIM2KO* MCF7 cells (Fig. 7c, d). The glutamate-derived antioxidant glutathione (GSH) showed increased isotopic enrichment from ^13^C-glutamine and overall abundance in *SIM2KO* cells (Fig. 7c, d). We postulate that increased ROS scavenging as a result of increased GSH formation protects the cell from ROS generated by defective MRCs. In support of this hypothesis, we measured mitochondrial superoxide levels after treating cells with MitoSOX stain and measured the relative fluorescence and found that MitoSOX fluorescence was lower in *SIM2KO* MCF7 cells than in control cells, indicating reduced mitochondrial superoxide levels (Fig. 7e). These data further show that glutaminolysis is essential to metabolic reprogramming as a result of dysfunctional electron transport due to impaired SC formation and contributes to the high-proliferation phenotype observed in *SIM2KO* cells. Indeed, cells with loss of *SIM2s* cultured in glutamine-free medium showed a lower proliferation rate than control cells (Fig. 7f, g)

## Discussion

**Fig. 8:**
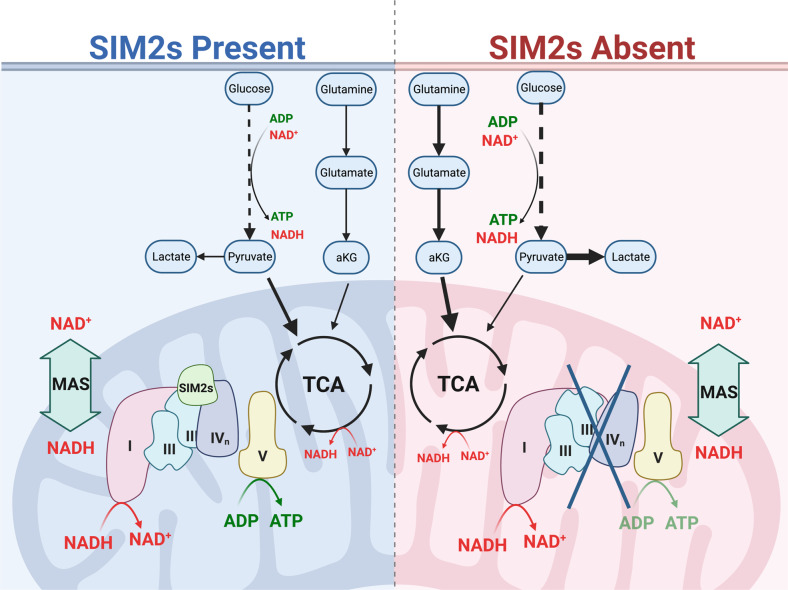
Diagram depicting the difference in metabolism with and without SIM2s. SIM2s promotes proper supercomplex formation and electron transport, leading to the mitochondrial production of ATP. When SIM2s is lost, mitochondrial production of ATP is reduced, and glycolytic production of ATP is needed. Additionally, glutaminolysis is elevated to provide cellular resources and support elevated glycolysis.

One of the recently discovered hallmarks of cancer is dysregulated cellular energetics and metabolic adaptation^67^. The idea that respiration impairment leads to aerobic glycolysis has been fundamental to our understanding of cancer metabolism^5^^18^.F-fluorodeoxyglucose positron emission tomography (FDG-PET), which is widely used to identify and track tumor cells, relies on the principle suggesting that cancer cells utilize large amounts of glucose for glycolysis^16,17^. Many studies have investigated this phenomenon in a variety of cancer types, and the results have revealed that multiple pathways coordinate to shift cell metabolism away from mitochondrial respiration and toward glycolysis^68–72^. Our findings highlight a newly identified factor, SIM2s, that regulates metabolic adaptation and demonstrates the complexity of the adaptation associated with mitochondrial supercomplex dynamics (Fig. 8).

Previous studies in our laboratory have shown that SIM2s is a tumor suppressor in breast cells, and we demonstrated that SIM2s suppressive action is in part mediated through the transcriptional repression of EMT factors and the NFkB pathway and that SIM2s promotes DNA damage repair through its direct interactions with ATM and the stabilization of replication forks^35,36,43,44,48^. In this study, we discovered a novel mechanism by which SIM2 suppresses tumor growth by directly interacting with MRCs to promote proper complex formation and SC assembly, leading to increased OXPHOS rates. We showed that SIM2s plays a key role in incorporating CIII subunits into CIII and SCs. Loss of SIM2s altered SC formation, compromising the transfer of electrons through the MRC, even though CI activity was retained. This finding supports a recently proposed “cooperativity model” since SCs were assembled despite the loss of SIM2s, but with reduced function owing to loss of these crucial subunits^33^. As a result, aerobic glycolysis provides ATP, and glutaminolysis provides glutamate, aKG, malate, and aspartate to facilitate NAD^+^ and NADH shuttling through the MAS. Consequently, additional cellular building blocks, such as nonessential amino acids (NEAAs) and nucleotides, are synthesized, allowing increased cell proliferation.

One of the biggest challenges in assessing and treating breast cancer, particularly ductal carcinoma in situ (DCIS), is tumor heterogeneity. In 2019, it was estimated that 41,760 women would be diagnosed with this preinvasive form of breast cancer, but as few as 20% of the DCIS cases will progress to invasive ductal carcinoma (IDC)^73,74^. Because these preinvasive lesions can potentially become dangerous, they are often treated through mastectomy or breast-conserving surgery followed by radiation and/or chemotherapy, leading to approximately 80% of patients with DCIS enduring needless destructive treatments^75,76^. Thus, the identification of predictive biomarkers is greatly needed. Despite monumental efforts to deconvolute intertumor and intratumor genomic heterogeneity to find biomarkers, no effective diagnostic markers to predict the tumors that will progress and those that will not have been identified. In our previous work, we observed that the SIM2s protein was stabilized in response to cellular stressors, including DNA damage and differentiation cues in the mammary gland^44^. Moreover, we showed that overall SIM2s protein expression in DCIS lesions was increased compared to that in normal tissue and that SIM2s was distributed throughout SIM2s-positive cells^38,44^. Notably, SIM2s was lost when a tumor progresses to IDC^38,44^. We postulate that SIM2s is increased in DCIS tissue and is increasingly localized to mitochondria, where it responds to harsh microenvironmental conditions, e.g., glucose deprivation, hypoxia, and increased acidification. As we have shown, cells with *SIM2s* loss show an increased demand for glucose and glutamine due to OXPHOS inhibition and thus outcompete other cells for nutrients. In addition, Damaghi et al. demonstrated that cells with a Warburg phenotype induced by microenvironmental changes showed transcriptional changes, suggesting that nutrient availability affects gene expression^77^. Therefore, in addition to direct SIM2s regulation of transcription, SIM2s-induced metabolic reprogramming can mediate changes to transcription.

Since their discovery, supercomplex functions have been debated. The most obvious advantage of SCs is an increase in the efficiency of electron transport substrate channeling to subsequent complexes. However, structural analysis by some research groups has indicated that the distance between the individual complexes in an SC is too great for substrate channeling to occur^78,79^. Studies have also indicated that substrate channeling is mediated by the formation of two distinct CoQ pools, one that depends on the oxidation of FADH_2_ and one that depends on the oxidation of NADH^23,26,80^. Our data support the idea that SC formation does not inherently promote electron transport but that the formation of SCs in certain structural arrangements, the formation of which is mediated by SIM2s, promotes OXPHOS. Similarly, the mitochondrion-stabilizing factor COX7A2L/COX7RP/SCAF1 promotes OXPHOS by altering the formation of SCs, allowing for substrate channeling^26,31^. Therefore, the proteins involved as well as the mechanism by which SCs are assembled are key to their ability to efficiently pass electrons^23,26,31^. Our data suggest that SIM2s supports OXPHOS independent of COX7A2L activity (data not shown); however, further analysis of SIM2s -mediated SC formation is necessary to confirm this finding. In addition to substrate channeling for efficient electron transport, SCs have been shown to support the functions of other pathways. Regardless of their increasing function, SCs have been shown to stabilize individual MRC complexes, in particular CI and CIII, as was apparent by the fact that approximately 90% of CI and 50% of CIII are found in SCs^18,21,27,81,82^. Additionally, the formation of SCs reduced ROS formation regardless of the ability of the SCs to transport electrons, and disassociation of SC I + III_2_ increased ROS levels^24,83^. Our data indicate that the formation of CI-containing SCs may be advantageous to a cell by reducing the generation of ROS. This idea and the increase in GSH level via glutaminolysis may explain the reason that *SIM2s* loss leads to decreased ROS production even when mitochondria are fragmented, which typically correlates with ROS production^84^.

One of the most interesting phenotypes we observed with loss of *SIM2s* is the decrease in mitochondrial ATP production but retention of SC formation and CI NADH oxidation. Conventional wisdom suggests that oxidation of NADH to generate only ROS would be detrimental to a cell, and thus, this process would be repressed^85^. However, our model revealed that increased NADH metabolism coupled with SC-mediated reduction in ROS production and an increase in ROS-scavenging pathway activity can maintain balanced pools of NAD^+^ and NADH to sustain pathways that depend on NAD^+^. These pathways include glycolysis, the pentose phosphate pathway (PPP), epigenetic regulation, and pathways involved in the formation of certain NEAAs; all of these pathways are fundamental to the Warburg phenotype. Moreover, in cells with aerobic glycolysis sustained with NADH oxidation by CI require increased glutaminolysis to maintain an active TCA cycle and support increased mitochondrial NAD^+^ export via the MAS.

Given that SC formation is a highly dynamic process and that the mechanism of SC assembly plays a major role in the function of cells, we hypothesize that SC regulation is a key factor in cell differentiation^18,21,27,81,82^. We and others have shown that SCs respond to metabolic stressors^31^. Since SIM2s is stabilized by cellular stressors, such as DNA damage and differentiation cues, we hypothesize that SC regulation is involved in many biological processes and is an underappreciated mechanism in cellular homeostasis. Interestingly, *SIM2* is overexpressed in Down syndrome, and many of the phenotypes associated with Down syndrome patients are due to mitochondrial dysfunction, including increased incidence of Alzheimer’s and Parkinson’s disease, heart defects, and type 2 diabetes; however, the gene(s) responsible has not been identified^86^. Moreover, further investigation into the mechanism of SIM2s -mediated SC formation may shed light on the degree to which the stability or flexibility of SCs changes in response to various stressors. Understanding this response may lead to the development of a diagnostic tool to predict SC changes in response to chemotherapy in breast cancer. Recent studies have shown mitochondria are vulnerable targets in treatment-resistant breast cancer^87,88^. However, OXPHOS inhibitors do not completely eliminate tumors, suggesting that SC changes may confer resistance^87,88^. Therefore, modulation of SC formation may be a viable strategy to target residual tumor cells.

## Supplementary information

Supplemental Material
