# Supplementary material for: Noncanonical role of singleminded-2s in mitochondrial respiratory chain formation in breast cancer
Source: Exp Mol Med. 2023 May 1;55(5):1046–63. doi: 10.1038/s12276-023-00996-0 (PMC10238511; doi:10.1038/s12276-023-00996-0)

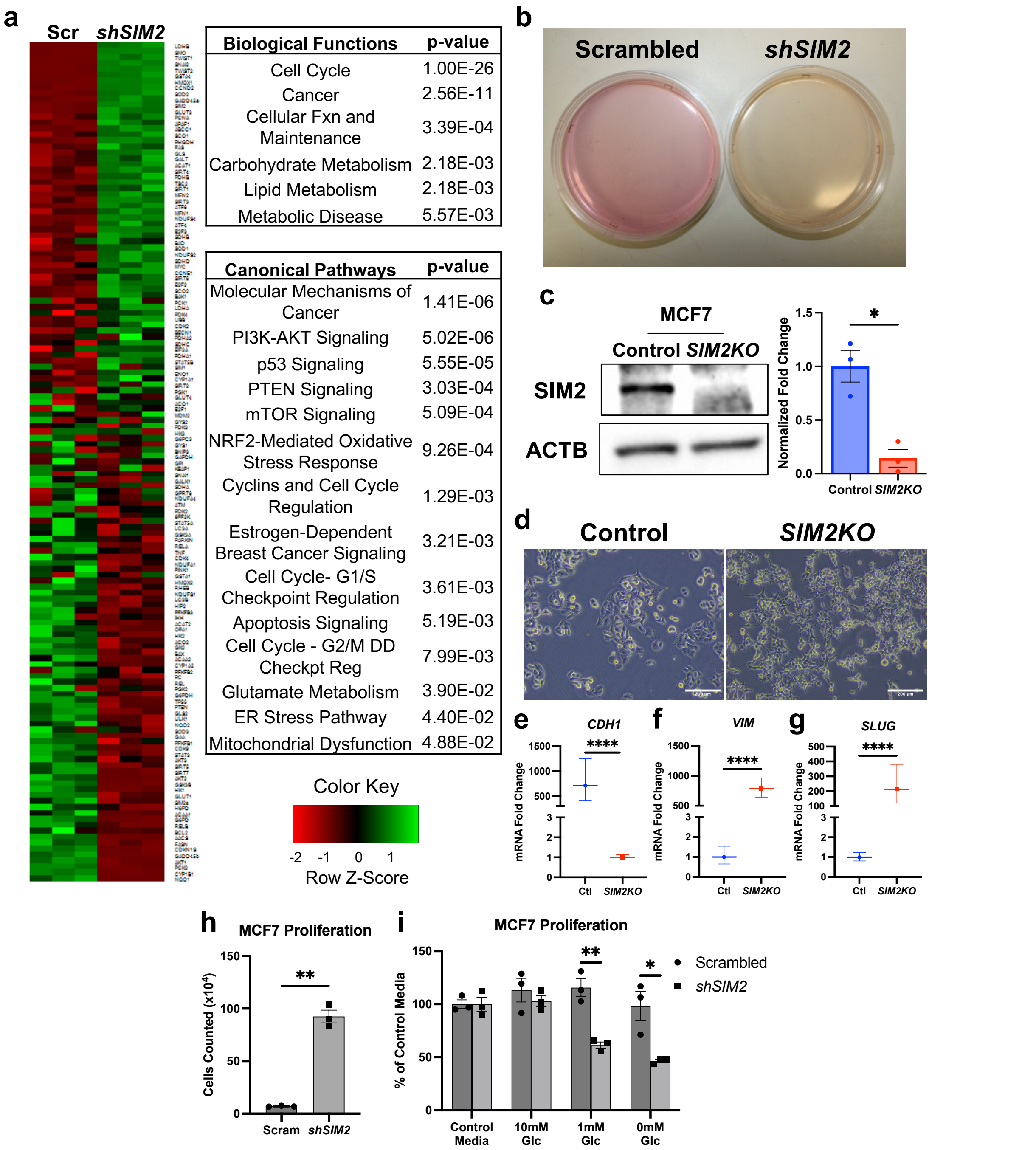

Supplementary Fig. 1 SIM2 loss leads to EMT and Warburg phenotype. **a** Microarray analysis of MCF7 *shSIM2* cells analyzed with Ingenuity Pathway Analysis. Significantly differentially regulated pathways are shown and have *P*<0.05. **b** Images of culture dishes with MCF7 scrambled control and *shSIM2* cells displaying different media color from changes in pH. **c** Immunoblot of SIM2 in MCF7 control and *SIM2KO* cells. Densiometric analysis were performed using Fiji and fold change of SIM2 normalized to ACTB is plotted showing means ± SEM. **P*<0.05, paired two-tailed t-test; *n=3*. **d** Brightfield image taken at 10X of MCF7 control and *SIM2KO* cells. **e-g** Quantitative RT-PCR analysis of MCF7 control and *SIM2KO* cells showing mRNA expression of **e** *CDH1*, **f** *VIM*, and **g** *SLUG*. Data is analyzed by the $2^{-\Delta\Delta Ct}$ method and error is presented as the transformed sum of squares of SD of the target and reference genes. *****P*<0.0001, two-tailed Student's *t* test of ΔCt values; *n*=3 samples. **h** Proliferation assays of MCF7 *shSIM2* cells in normal media. **i** MCF7 *shSIM2* cells were also grown in media containing 10mM, 1mM, and 0mM glucose and growth was represented as a percentage of growth in control media. Cells were counted 3 days after plating and comparisons were made to control cells at the given conditions. Data are presented as means ± SEM. **P*<0.05, ***P*<0.01, *****P*<0.0001, two-tailed Student's *t* test; *n*=3 samples.

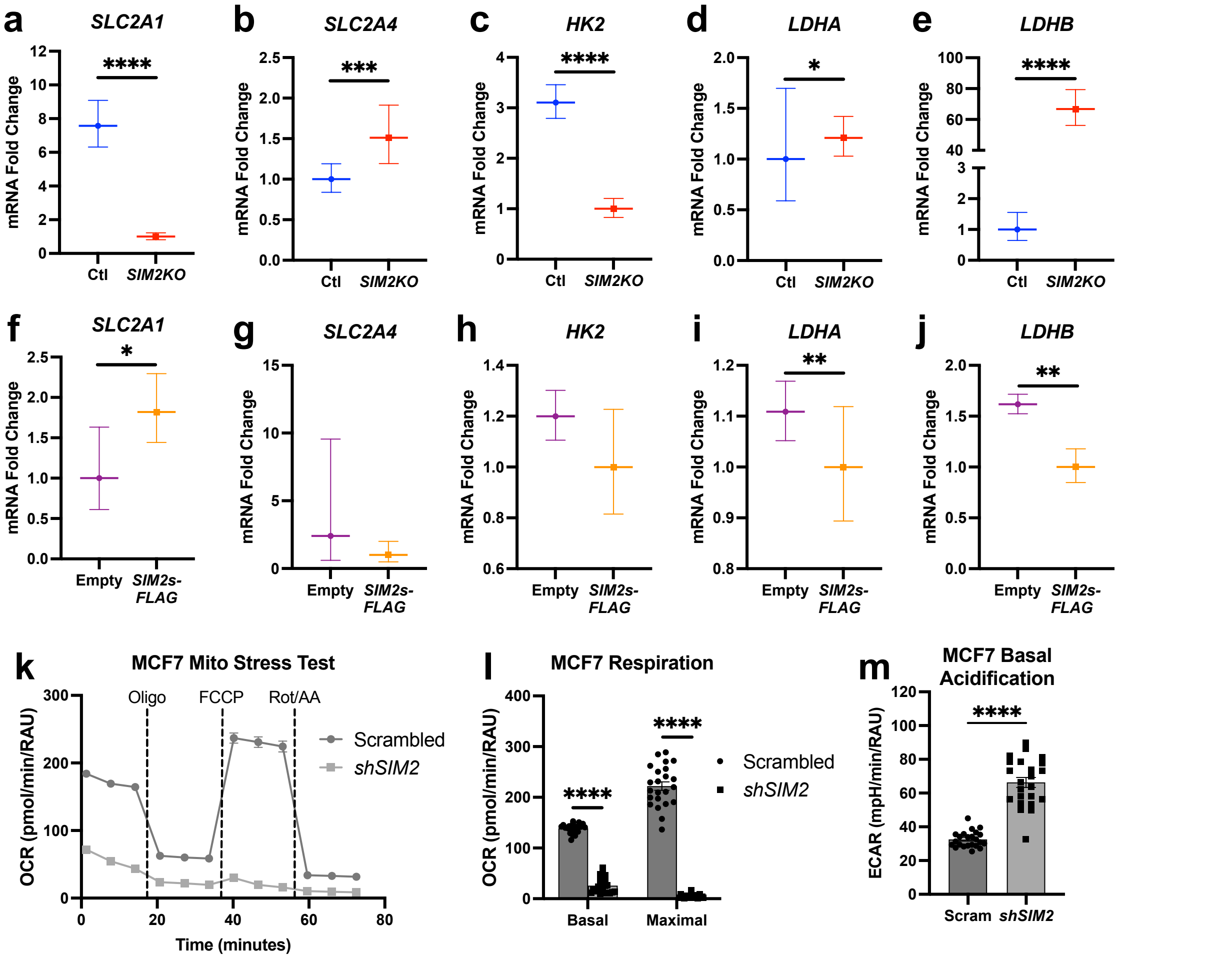

Supplementary Fig. 2 Glycolytic genes are not regulated by SIM2. **a-j** Quantitative RT-PCR analysis of **a-e** MCF7 *SIM2KO* and **f-j** SUM159 *SIM2s-FLAG* cells showing relative mRNA expression of *SLC2A1*, *SLC2A4*, *HK2*, *LDHA*, and *LDHB*. Data is analyzed by the $2^{-\Delta\Delta Ct}$ method and error is presented as the transformed sum of squares of SD of the target and reference genes. **P*<0.05, ***P*<0.01, ****P*<0.001, *****P*<0.0001, two-tailed Student's *t* test of ΔCt values; *n*=3 samples. **k-m** Mitochondrial stress test was performed using Seahorse XF Extracellular Flux Analyzer on MCF7 *shSIM2*. **k** Oxygen consumption rates (OCR) were plotted through the experiment. **l** OCR after rotenone/antimycin-A treatment was subtracted the third measurement and seventh measurement to obtain basal and maximal respiration rates, respectively. **m** Basal extracellular acidification rates (ECAR) were measured. Data are plotted as mean ± SEM. *****P*<0.0001, two-tailed Student's *t* test; *n*=23.

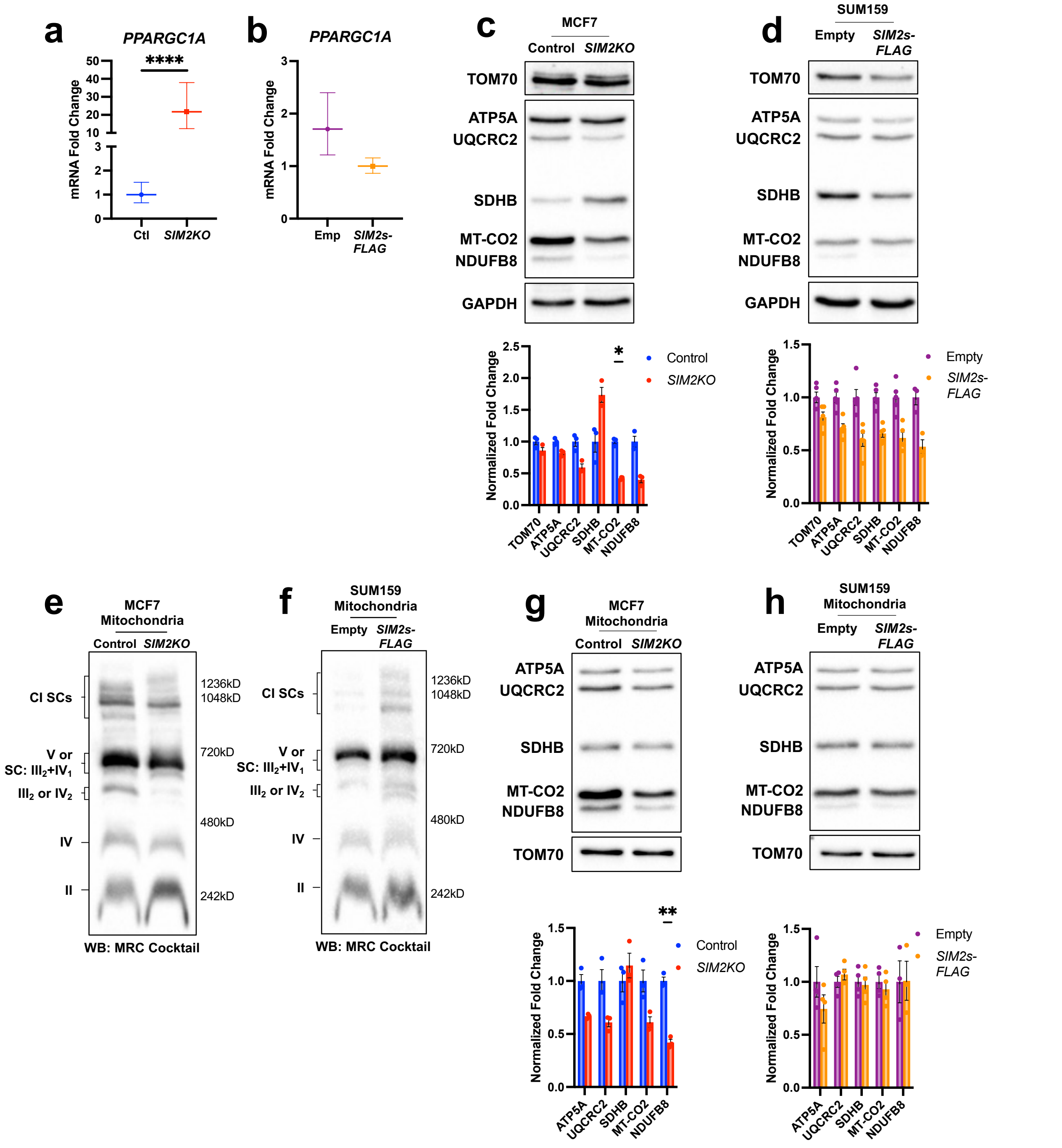

Supplementary Fig. 3 Mitochondrial protein expression is not correlated with SIM2 expression. **a-b** Quantitative RT-PCR analysis of **a** MCF7 *SIM2KO* and **b** SUM159 *SIM2s-FLAG* cells showing relative mRNA expression of *PPARGC1A*. Data is analyzed by the $2^{-\Delta\Delta Ct}$ method and error is presented as the transformed sum of squares of SD of the target and reference genes. ****$P<0.0001$, two-tailed Student's t test of ΔCt values; $n=3$ samples. Protein from **c** MCF7 *SIM2KO* and **d** SUM159 *SIM2s-FLAG* was analyzed by immunoblot using the Total OXPHOS cocktail (ATP5A, UQCRC2, SDHB, MT-CO2, NDUFB8), TOM70, and GAPDH antibodies. Densiometric analysis were performed using Fiji and fold change of each protein normalized to GAPDH is plotted showing means ± SEM. *$P<0.05$, paired two-tailed t-test; $n\geq3$. **e,f** Mitochondrial proteins from **e** MCF7 *SIM2KO* cells and **f** SUM159 *SIM2s-FLAG* cells were solubilized with 1:8 protein to digitonin and underwent BN-PAGE followed by immunoblot analysis using the Total OXPHOS cocktail antibody. Mitochondrial protein from **g** MCF7 *SIM2KO* and **h** SUM159 *SIM2s-FLAG* was analyzed by immunoblot using Total OXPHOS cocktail and TOM70 antibodies. Densiometric analysis were performed using Fiji and fold change of each protein normalized to GAPDH is plotted showing means ± SEM. *$P<0.05$, paired two-tailed t-test; $n\geq3$.

Supplementary Table 1 List of mitochondrial proteins found to interact with SIM2.

Gene Name	Protein	# of Peptides
HSPA1A	Heat shock 70kDa protein 1A	7
HADHB	Isoform 2 of trifunctional enzyme subunit beta, mitochondrial	6
GOT2	Aspartate aminotransferase, mitochondrial	5
ACADVL	Very long-chain specific acyl-CoA dehydrogenase, mitochondrial	5
RACK1	Receptor of activated protein C kinase	4
DECR1	Isoform 2 of 2,4-dienoyl-CoA reductase, mitochondrial	3
SQRDL	Sulfide:quinone oxidoreductase, mitochondrial	2
NDUFB10	Isoform 2 of NADH dehydrogenase [ubiquinone] 1 beta subcomplex subunit 10	2

Supplementary Methods

Gene Expression Microarray Analysis

The Whole Human Genome CodeLink Bioarray (Applied Microarrays) was used to determine differential gene expression between scrambled and *shSIM2* MCF7 breast cancer cells. Sample preparation and hybridization were performed according to the manufacturer's protocols and as previously described[38]. RNA samples were confirmed for loss of *SIM2s* expression via qPCR prior to microarray analysis. Raw microarray data were initially normalized and analyzed using CodeLink Expression Analysis Software version 4.1.0.29054 (GE Healthcare). A median normalization method was used, with a 20% threshold trim percentage. Microarray data were analyzed, and functionally analyses were generated through the use of Ingenuity Pathways Analysis (Ingenuity Systems). A data set containing gene identifiers and corresponding expression values was uploaded into the application. The identifiers were mapped to their corresponding objects in Ingenuity's Knowledge Base. A *P* value cutoff of 0.05 was set to identify the expression of which pathway was significantly differentially regulated. These molecules were integrated into a molecular network developed from information in Ingenuity's Knowledge Base. The data were then analyzed for the biological functions and/or diseases that were most significant. The data discussed in this publication have been deposited in NCBI's Gene Expression Omnibus and are accessible through GEO Series accession number GSE44187[39].

High Pressure Liquid Chromatography Mass Spectrophotometry

SUM159 pLPCX Empty control and *SIM2s-FLAG* samples were prepared by following the Co-immunoprecipitation until the addition of β-mercaptoethanol. The samples were further prepared using S-Trap micro spin columns (Protifi). The samples were then analyzed by capillary HPLC-electrospray ionization tandem mass spectrometry on an Orbitrap Velos Pro mass spectrometer (Thermo Fisher) fitted with a New Objective Digital PicoView 550 NanoESI

source. On-line HPLC separation was accomplished with an Eksigent/AB Sciex NanoLC-Ultra 2-D HPLC system: column, PicoFrit™ (New Objective; 75 µm i.d.) packed to 15 cm with C18 adsorbent (Vydac; 218MS 5 µm, 300 Å); mobile phase A, 0.5% acetic acid (HAc)/0.005% trifluoroacetic acid (TFA); mobile phase B, 90% acetonitrile/0.5% HAc/0.005% TFA; gradient 2 to 42% B in 30 min; flow rate, 0.4 µl/min. MS conditions were: ESI voltage, 2.75 kV; MS1 scan range, m/z 300 - 2000; isolation window for MS/MS, 3; normalized collision energy, 30%; scan strategy, survey scan followed by acquisition of data dependent collision-induced dissociation (CID) spectra of the six most intense ions in the survey scan above a threshold of 3000; dynamic exclusion, 30 sec; no charge state screening; reject list of background ions (± 10 ppm). Mascot (Matrix Science, version 2.5.1) was used to search the spectra against a combination of the following databases: UniProt_Human_20161004 (92,910 sequences; 36,855,958 residues); common contaminants (247 sequences; 12,813 residues). Peptide tolerance was 20 ppm, fragment tolerance, 0.8 Da. Cysteine carbamidomethylation was set as a fixed modification and methionine oxidation, deamidation of glutamine and asparagine and protein N-terminal acetylation were considered as variable modifications; trypsin was specified as the proteolytic enzyme, with one missed cleavage allowed. Subset searches of the identified proteins by X! Tandem [CYCLONE (2010.12.01.1)], cross-correlation with the Mascot results and determination of protein and peptide identity probabilities were accomplished by Scaffold (Proteome Software, version 4.7.1). The thresholds for acceptance of peptide and protein assignments in Scaffold were 95% and 99%, respectively with a minimum of two peptide required, resulting in a protein-level FDR of 0%

Supplementary Table 2 Details of antibodies used in experiments.

Immunoblot Antibodies	Source	Cat. No.	Dilution
SIM2	Abcam	ab131161	1:1000
SIM2 (BNPAGE and CoIP)	Aviva Systems Biology	ARP38551_P050	1:500
Beta-Actin	Cell Signaling	3700	1:2000
Total Oxphos Human Cocktail	Abcam	ab110411	1:250
TOM70	Proteintech	14528-1-AP	1:1000
NDUFS2	Abcam	ab110249	1:1000
UQCRC2	Abcam	ab14745	1:1000
UQCRFS1	Abcam	ab14746	1:1000
MT-CO2	Abcam	ab110258	1:1000
Alpha-tubulin	Santa Cruz	sc8035	1:200
PARP1	Cell Signaling	9542	1:1000
VDAC1	Abcam	ab14734	1:1000
NDUFB10	Invitrogen	MA5-26082	1:500
OPA1	BD Biosciences	612607	1:1000
DRP1	Millipore	ABT155	1:1000
pDRP1(S673)	Cell Signaling	4867S	1:1000
LDHA	Proteintech	21799-1-AP	1:500
MDH1	Proteintech	15904-1-AP	1:5000
PDH	Cell Signaling	3205P	1:1000
OGDH	Proteintech	15212-1-AP	1:5000
Anti-Mouse HRP (Secondary)	Cell Signaling	7076	1:5000
Anti-Rabbit HRP (Secondary)	Cell Signaling	7074	1:5000
**Proximity Ligation Antibodies**	**Source**	**Cat. No.**	**Dilution**
FLAG	Cell Signaling	14793	1:800
NDUFB10	Invitrogen	MA5-26082	1:100
UQCRC2	Abcam	ab14745	1:2000
MT-CO2	Abcam	ab110258	1:500

Supplementary Table 3 Primer sequences used for qRT-PCR analysis.

Primer Pair	Forward 5'-3'	Reverse 5'-3'
CDH1	CACAGACGCGGACGATGAT	GATCTTGGCTGAGGATGGTGTAA
ESR1	TCTGCCAAGGAGACTCGCTACT	CGTTATGTCCTTGAATACTTCTCTTGA
VIM	TTCTCTGCCTCTTCCAAACTTTTC	GGGTATCAACCAGAGGGAGTGA
SLUG	GGCTGGCCAAACATAAGCA	CCTTGTCACAGTATTTACAGCTGAAAG
SLC2A1	CAGCTGACGTGACCCATGAC	CCTTCTTCTCCCGCATCATC
SLC2A4	GCTTCGTGGCATTTTTTGAGA	ACGTCGGCCACGATGAAC
HK2	GCATCTTTGAAACCAAGTTCTTGTC	GGTGCTCTCAAGCCCTAAGTGT
LDHA	AAATTGAAGGGAGAGATGATGGAT	AGTTACATTATAGTCTTTGCCAGAGA
LDHB	GGGAACATGGCGACTCAAGT	GAGAAACACCTGCCACATTCAC
PPARGC1A	TGTCACCACCCAAATCCTTATTT	TGTGTCGAGAAAAGGACCTTGA
NDUFB10	TACCAGGACCGCTATCAGGA	CGGCCTCTTTTGCAGCTTTT
COX7A2L	GCCTGCCTGACCAAATGC	GGGCGATCAGGCAGT
SCO2	CTTCACTCACTGCCCTGACA	TGAGCAGGTAGATGGCAATG
OPA1	GTGGTTGGAGATCAGAGTGCTG	GAGGACCTTCACTCAGAGTCAC
DNM1L	GATGCCATAGTTGAAGTGGTGAC	CCACAAGCATCAGCAAAGTCTGG
MDH2	GGAGTGGCCGCAGATCTG	TCAGGTCCGAGGTAGCCTTTC
GAPDH	CCAGGTGGTCTCCTCTGACTTC	GTGGTCGTTGAGGGCAATG
TBP	TGCACAGGAGCCAACAGTGAA	CACATCACAGCTCCCCACCA
ACTB	GTTTGAGACCTTCAACACCCC	GTGGCCATCTCTTGCTCGAAGTC

Supplemental Figure 1c

Legend
M7: MCF7
MKO: MCF7 *SIM2KO*

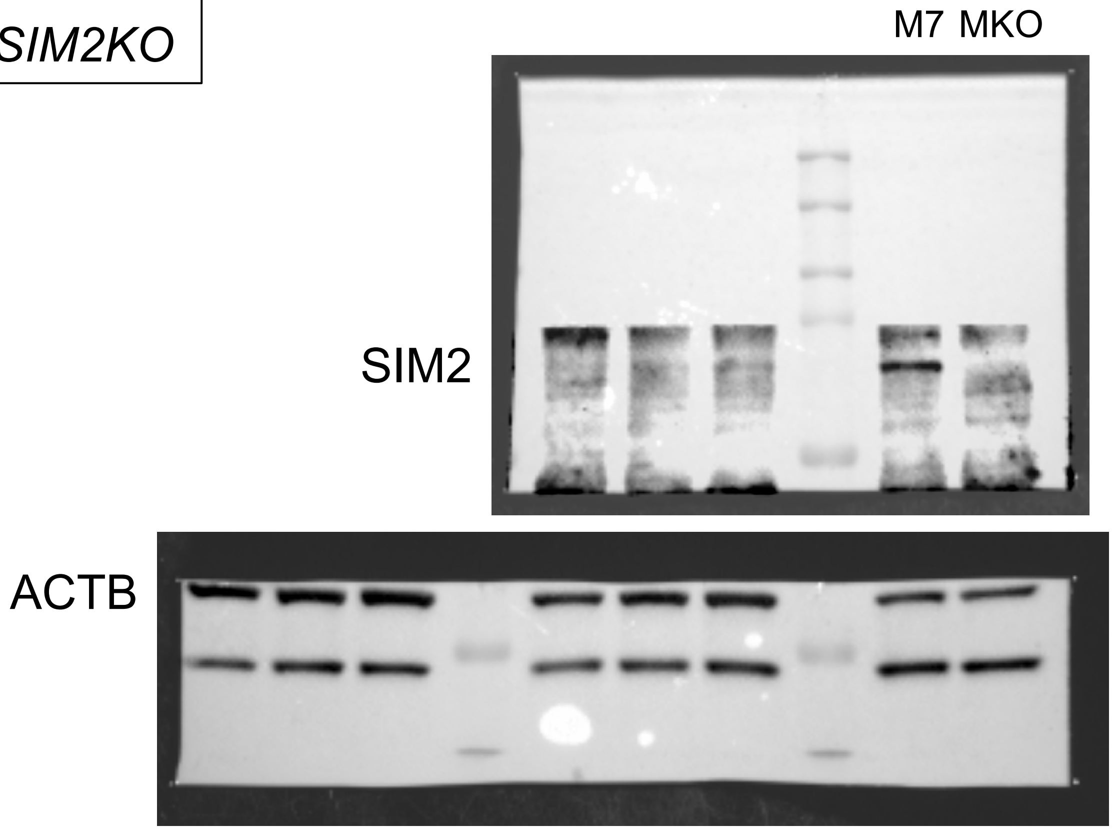

Supplemental Figure 3c

Legend
M7: MCF7
MKO: MCF7 *SIM2KO*

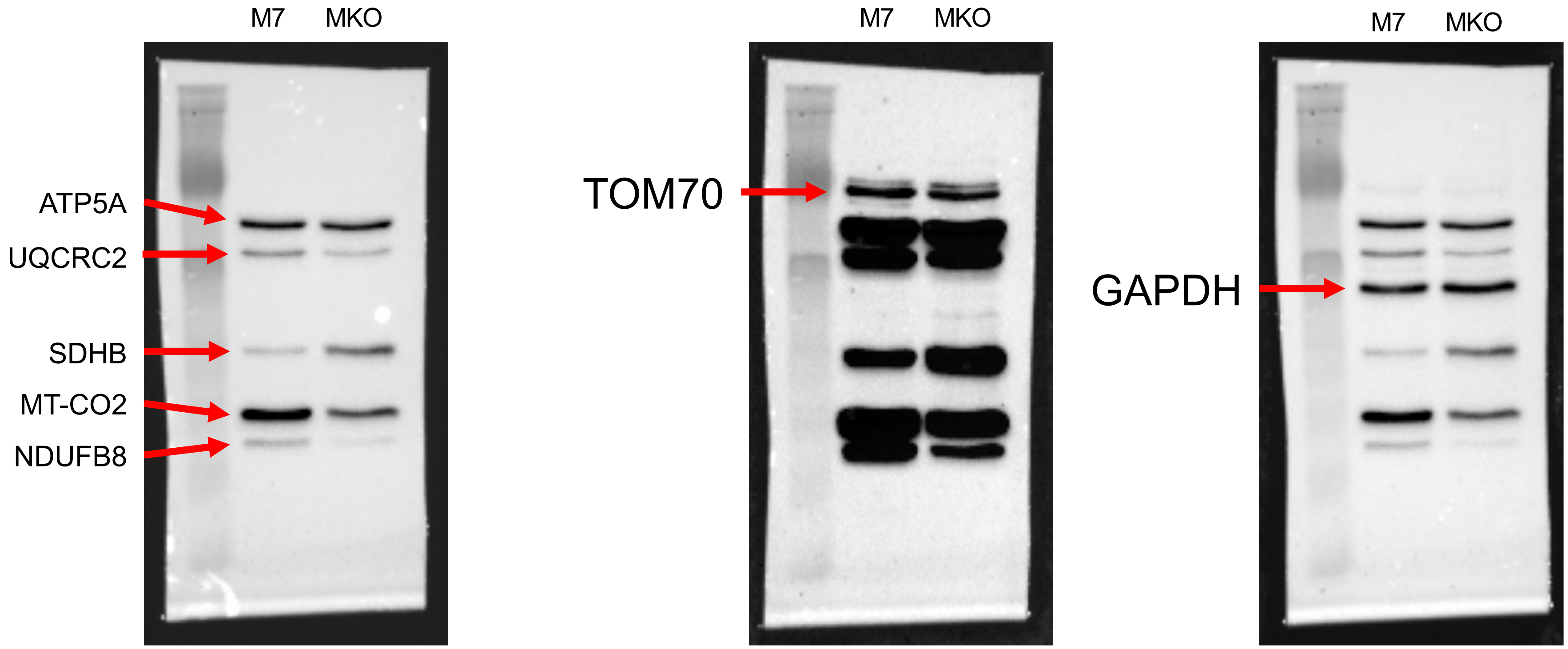

Supplemental Figure 3d,g,h

<u>Legend</u>
M7: MCF7
MKO: MCF7 *SIM2KO*
SE: SUM159 *Empty*
SO: SUM159 *SIM2s-FLAG*

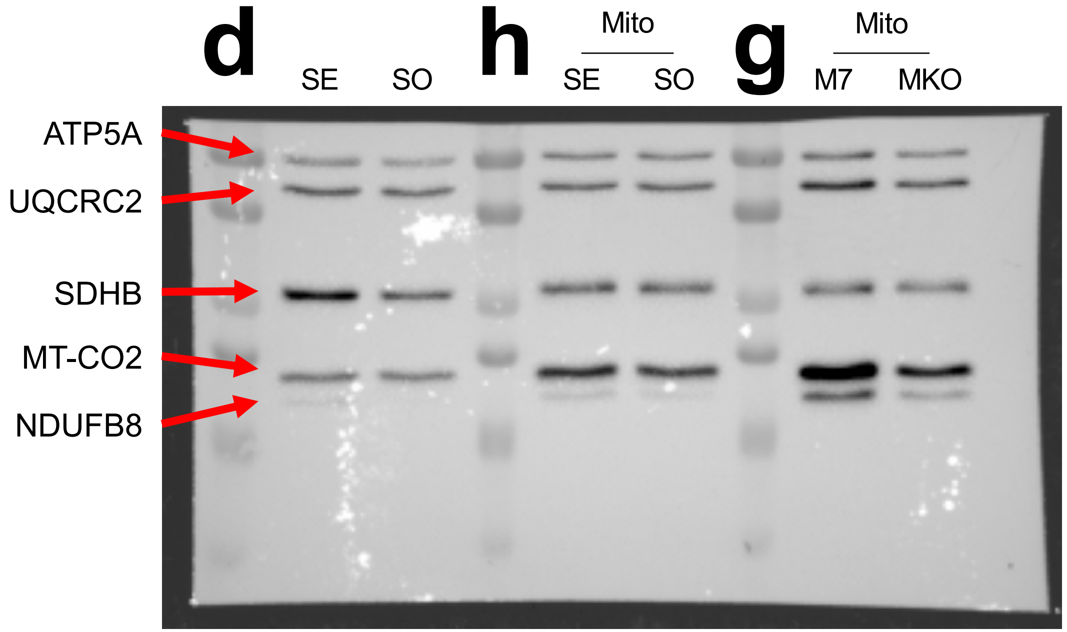

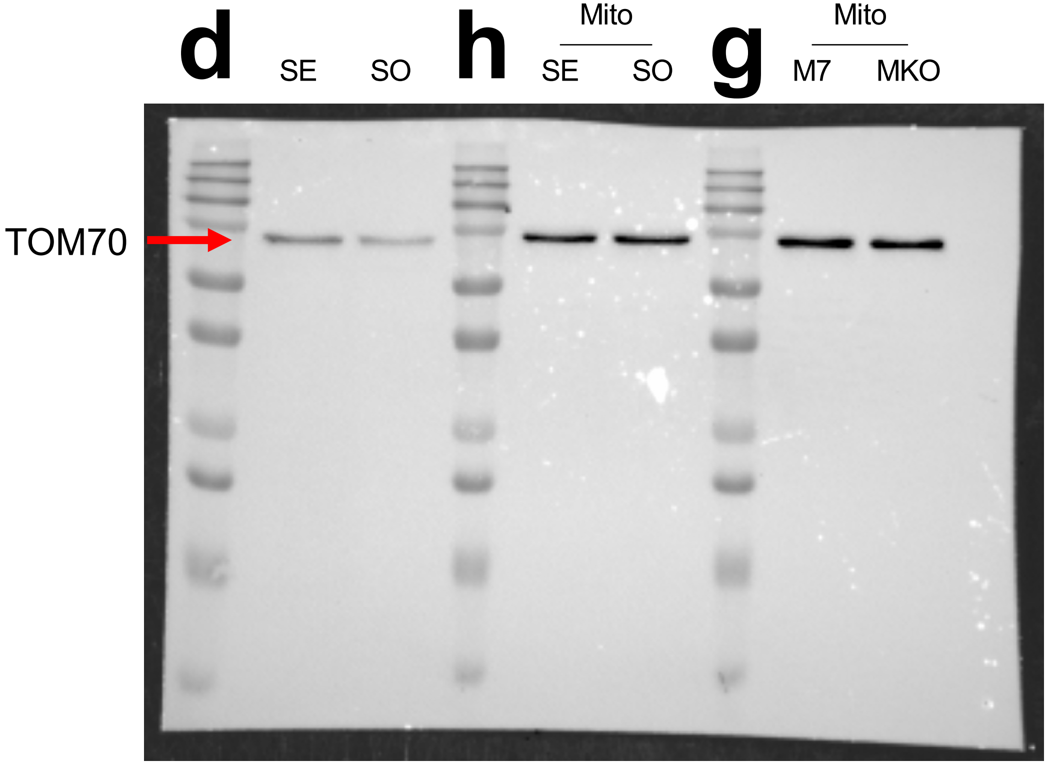

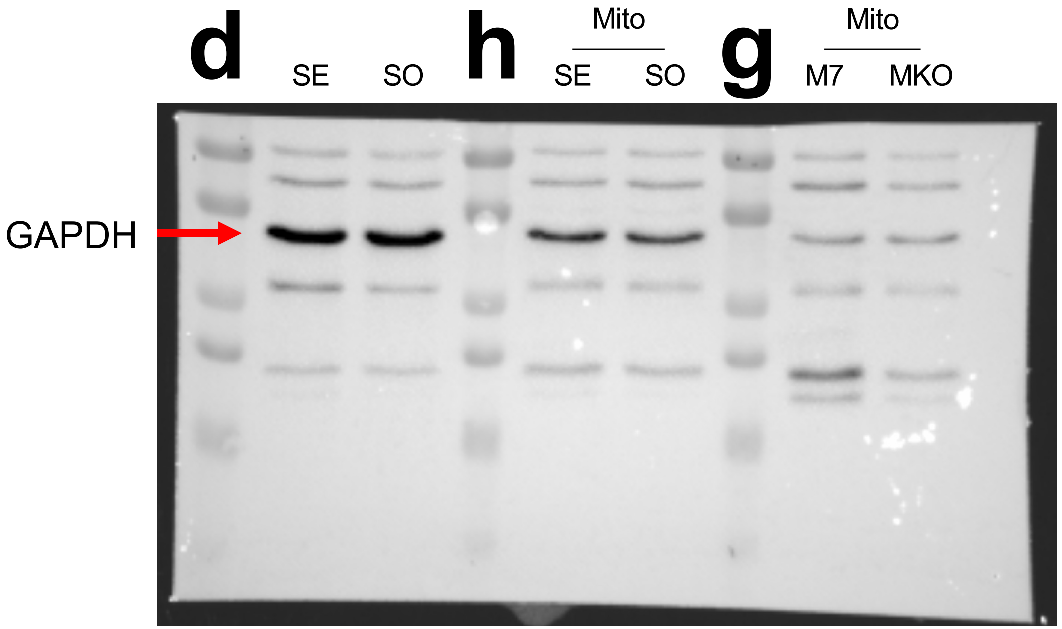

Supplemental Figure 3e,f

Legend
M7: MCF7
MKO: MCF7 *SIM2KO*
SE: SUM159 *Empty*
SO: SUM159 *SIM2s-FLAG*

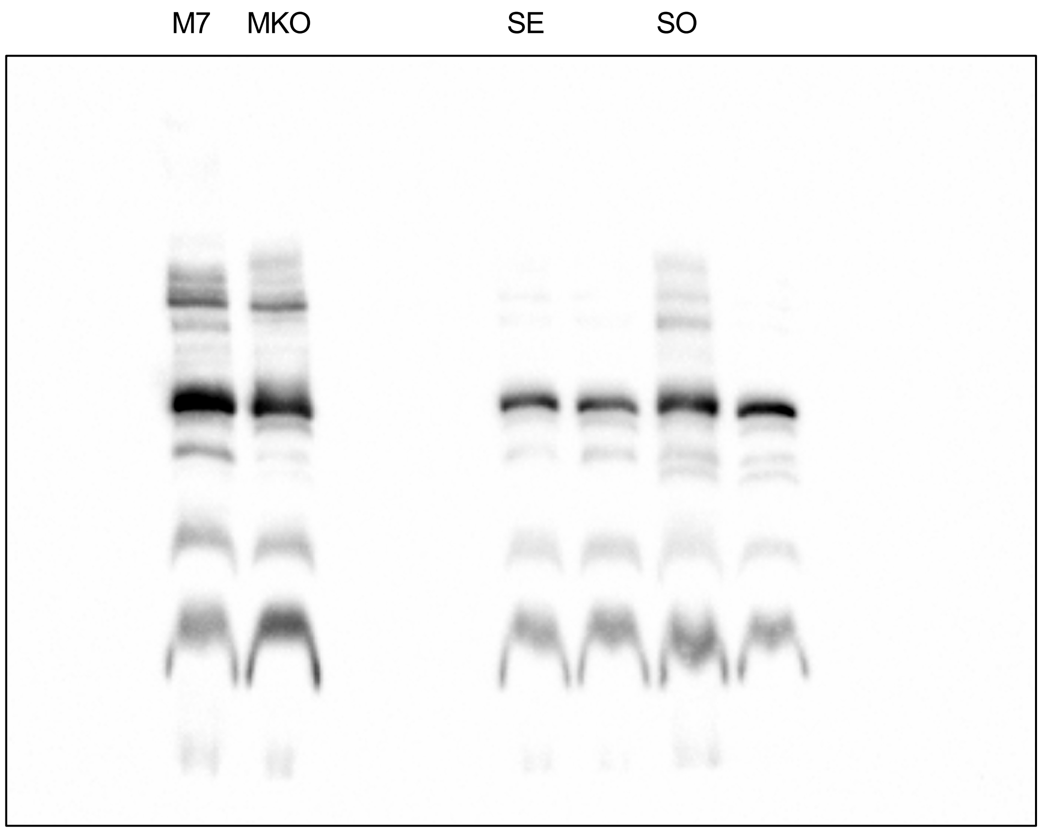

Total OXPHOS Cocktail

Figure 2a,b,c,d

<u>Legend</u>
M7: MCF7
MKO: MCF7 *SIM2KO*
SE: SUM159 *Empty*
SO: SUM159 *SIM2s-FLAG*

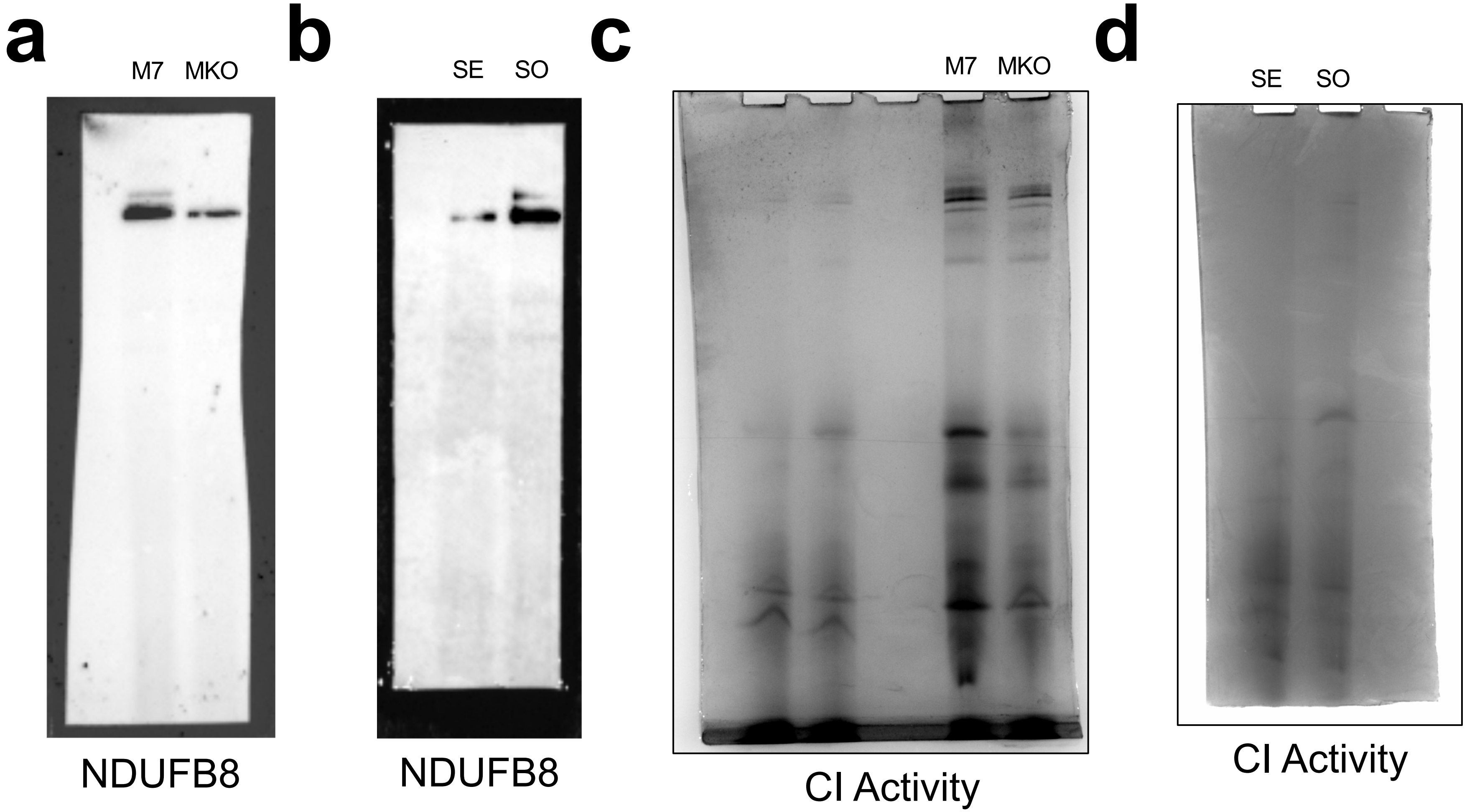

Figure 2e,f

Legend
M7: MCF7
MKO: MCF7 *SIM2KO*
SE: SUM159 *Empty*
SO: SUM159 *SIM2s-FLAG*

e

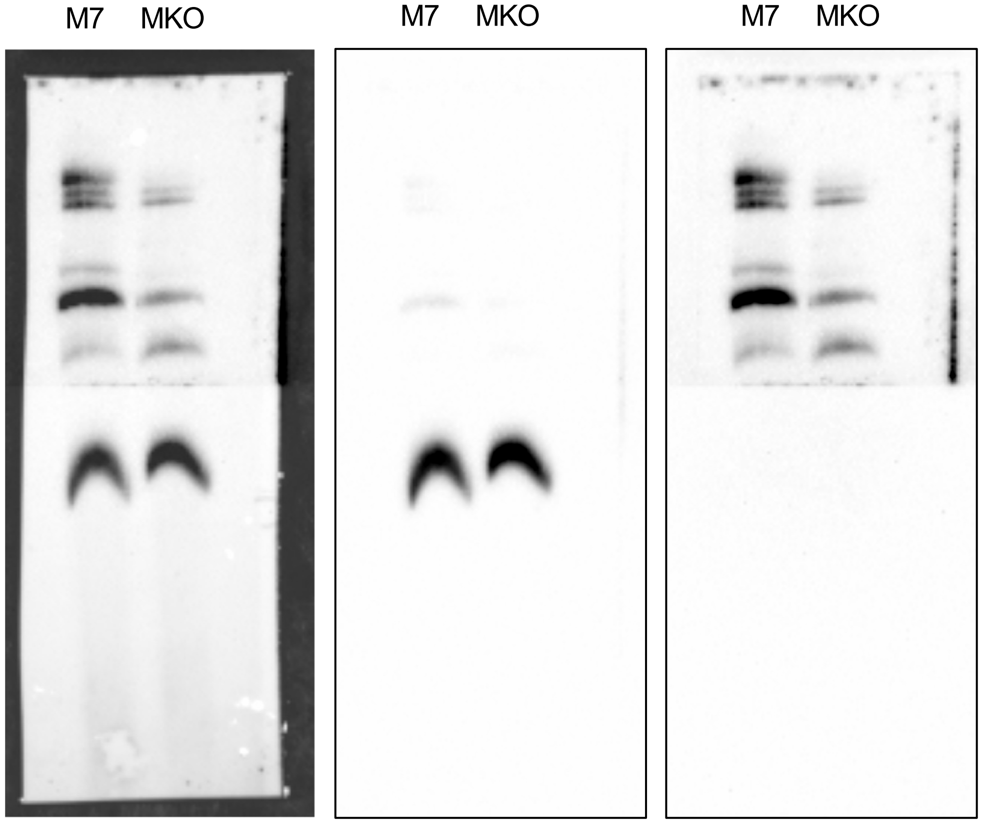

MT-CO2

f

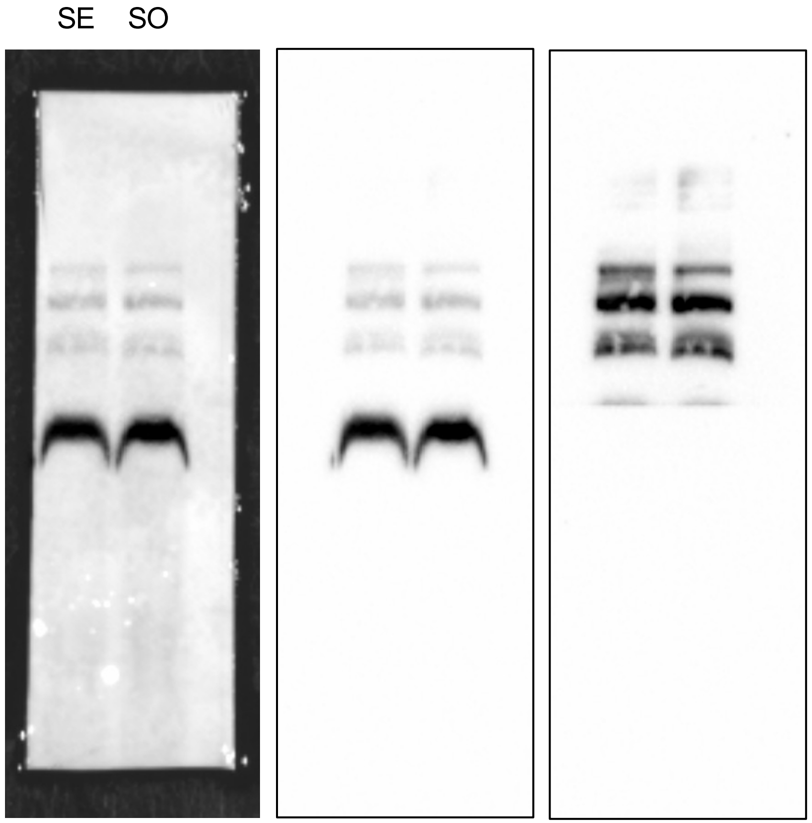

MT-CO2

Figure 2g,h,i,j

<u>Legend</u>
M7: MCF7
MKO: MCF7 *SIM2KO*
SE: SUM159 *Empty*
SO: SUM159 *SIM2s-FLAG*

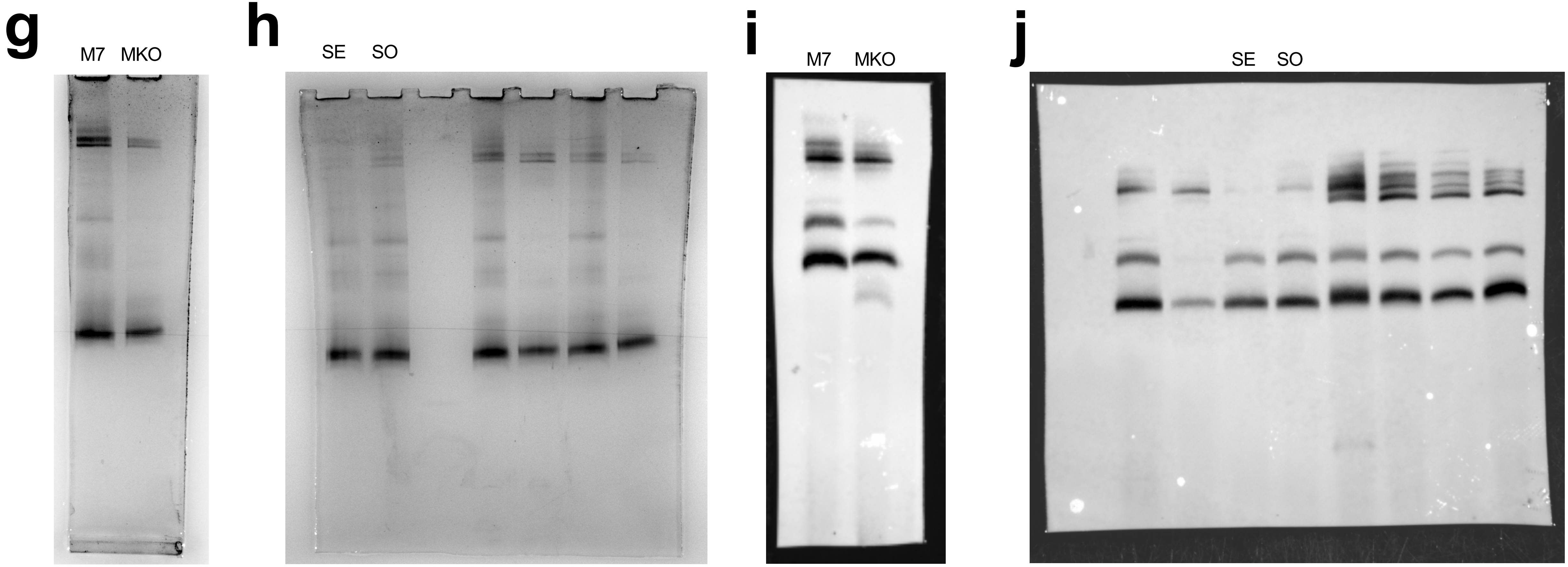

CIV Activity | CIV Activity | UQCRC2 | UQCRC2

Figure 2k,l

Legend
M7: MCF7
MKO: MCF7 *SIM2KO*
SE: SUM159 *Empty*
SO: SUM159 *SIM2s-FLAG*

k

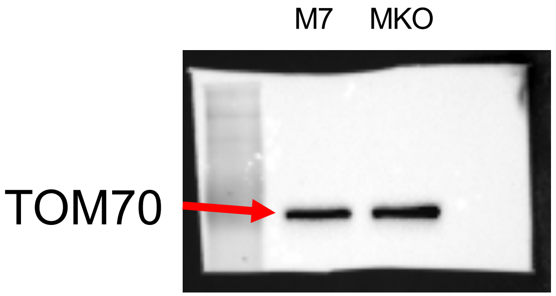

l

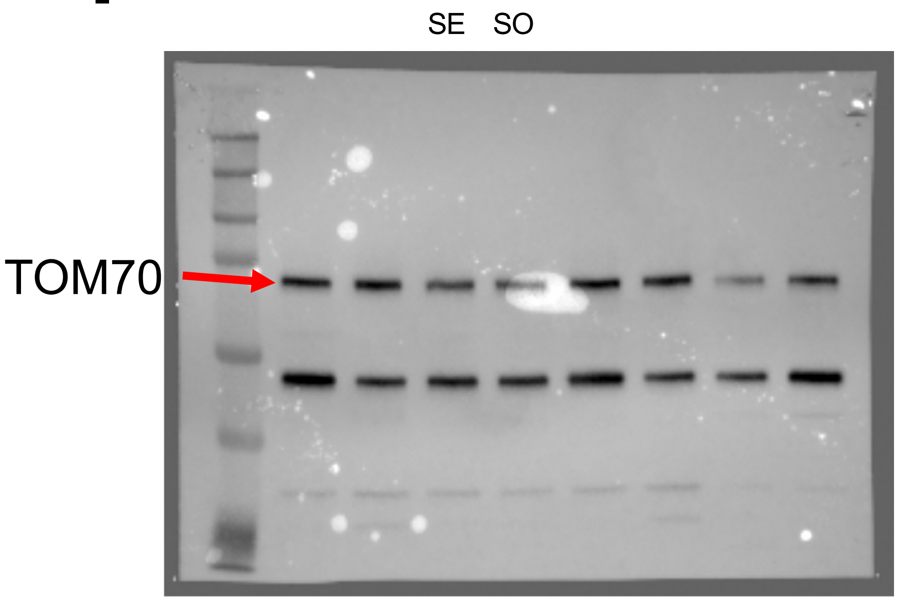

Figure 3a,c

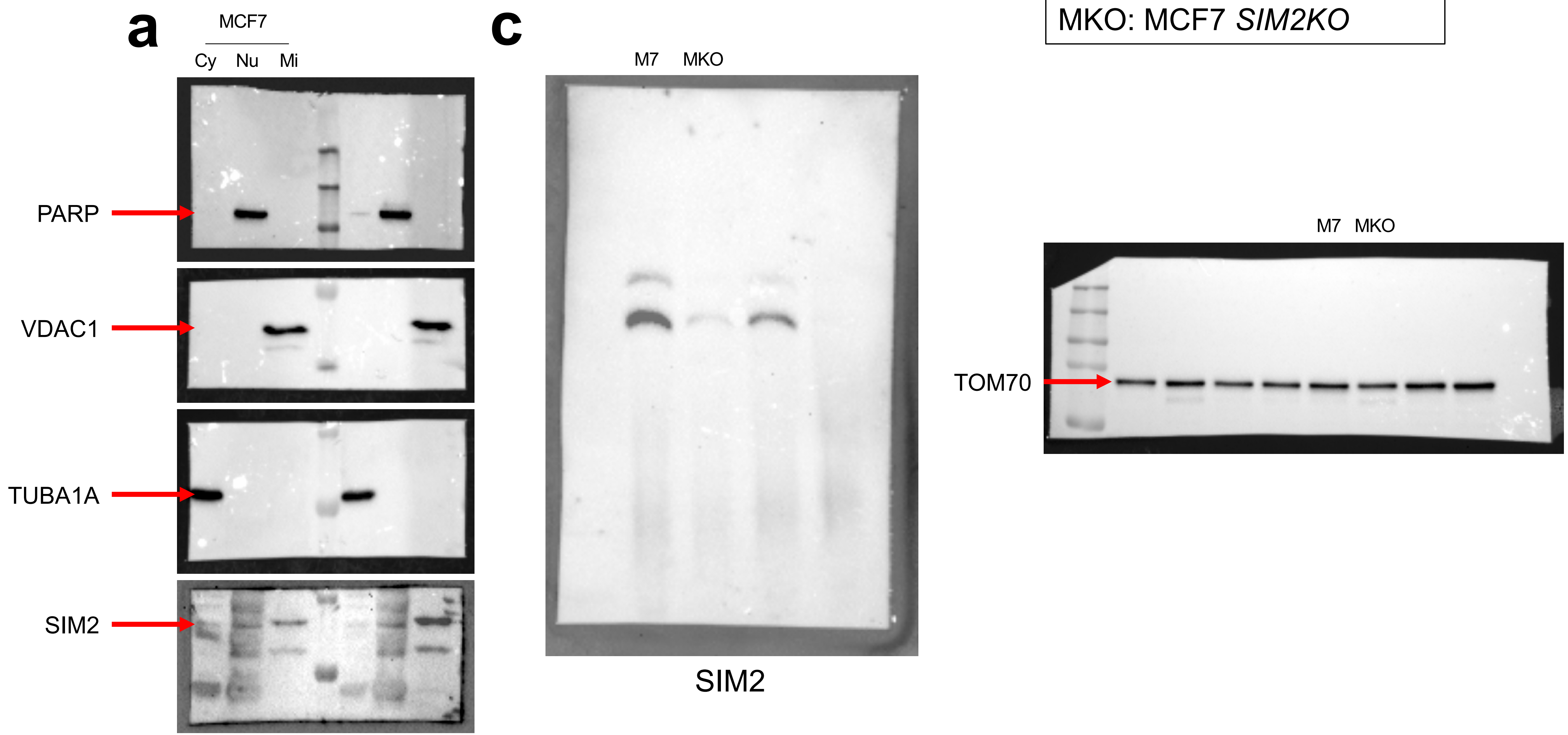

Legend
M7: MCF7
MKO: MCF7 *SIM2KO*

Figure 3e

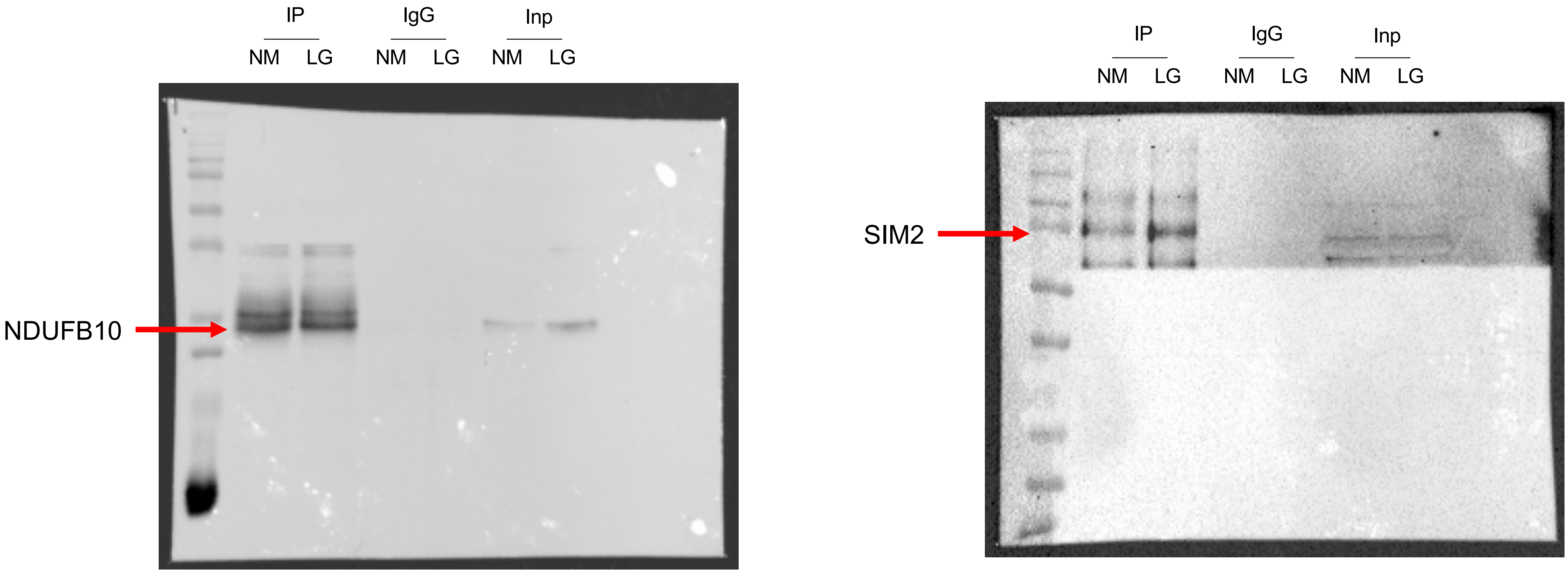
IP
IgG
Inp
NM
LG
NM
LG
NM
LG
NDUFB10
IP
IgG
Inp
NM
LG
NM
LG
NM
LG
SIM2

Figure 4a

Legend
M7: MCF7
MKO: MCF7 *SIM2KO*

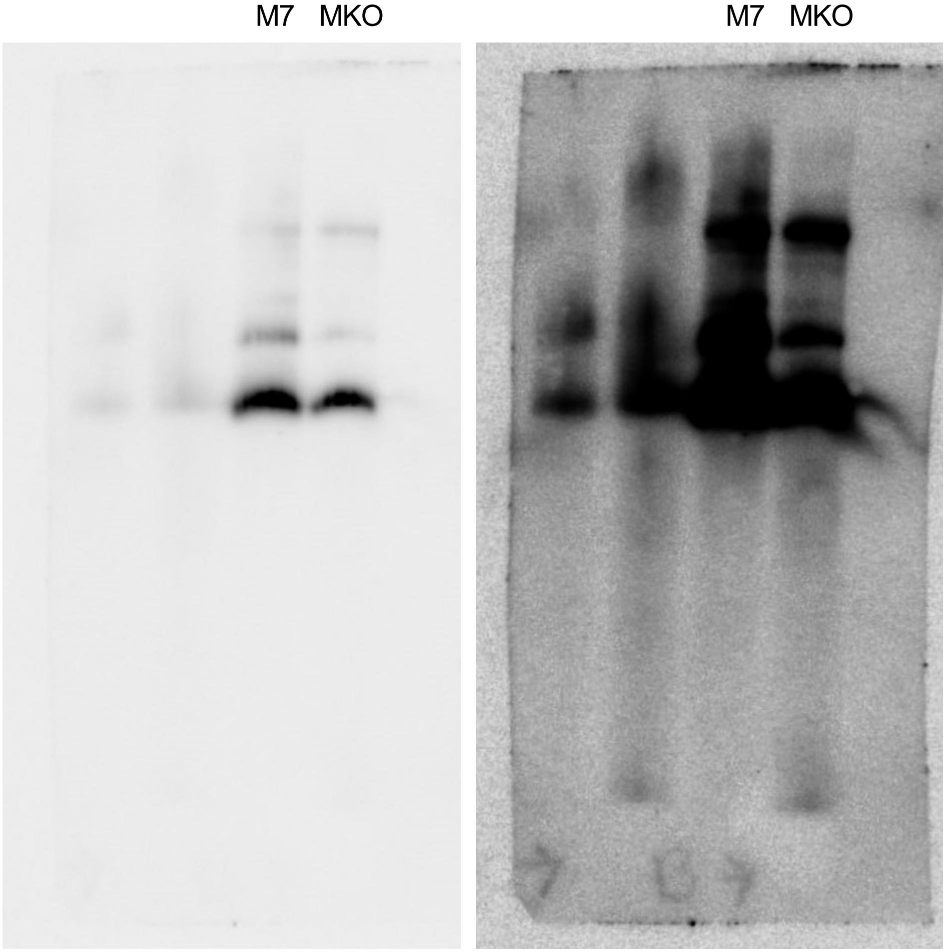

UQCRFS1

Figure 4b

Legend
M7: MCF7
MKO: MCF7 *SIM2KO*

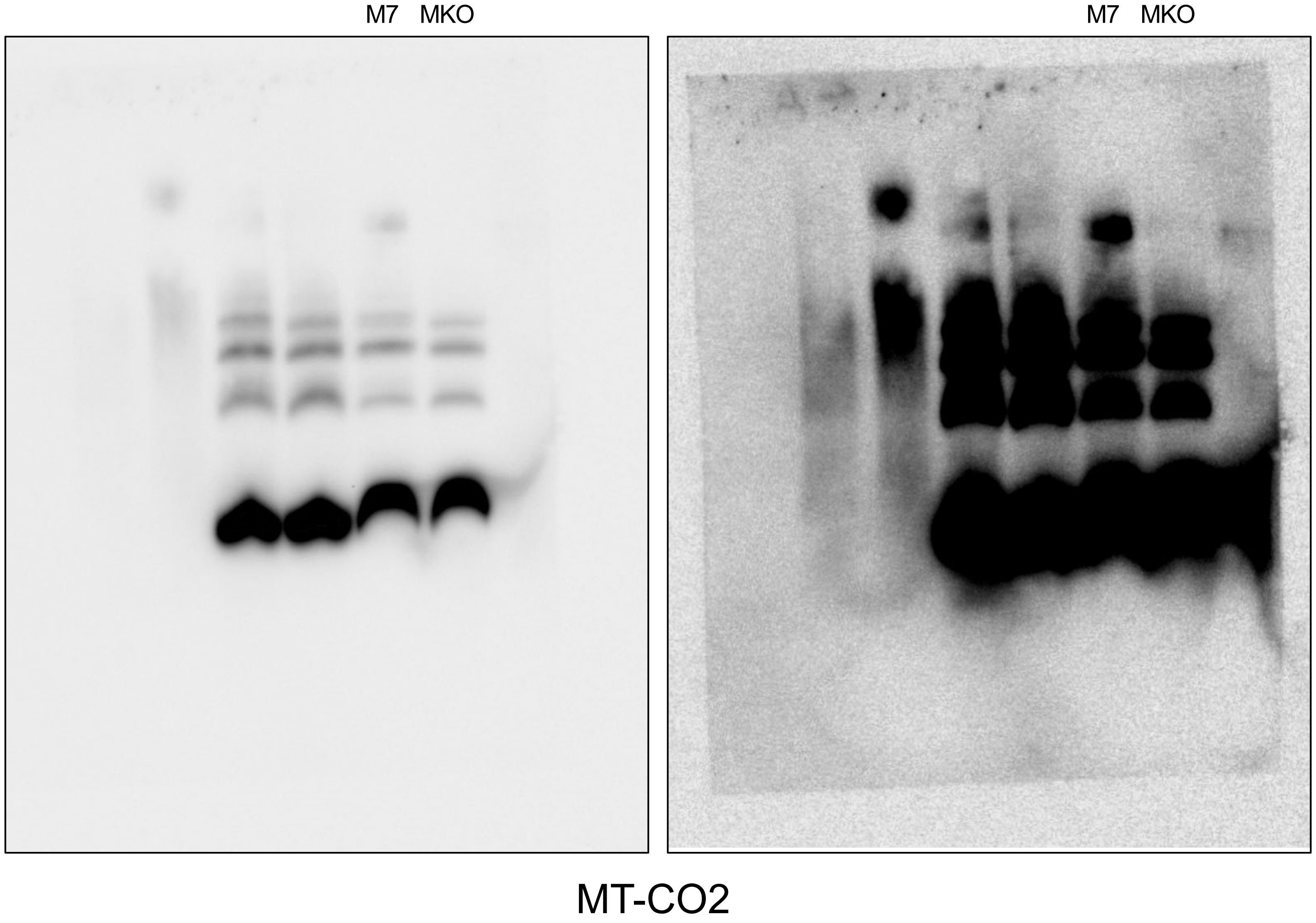

Figure 4c,d

Legend
siScr: MCF7 siRNA Scrambled
siCIII.1: MCF7 siRNA *UQCRC2* (#1)
siCIII.2: MCF7 siRNA *UQCRC2* (#2)

c

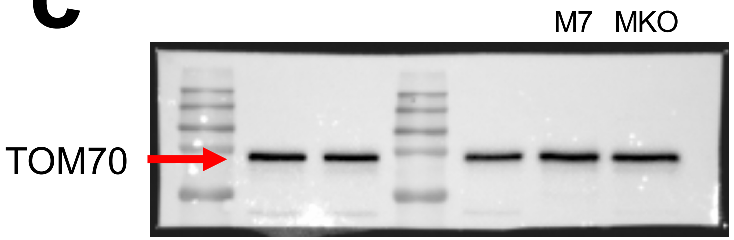

d

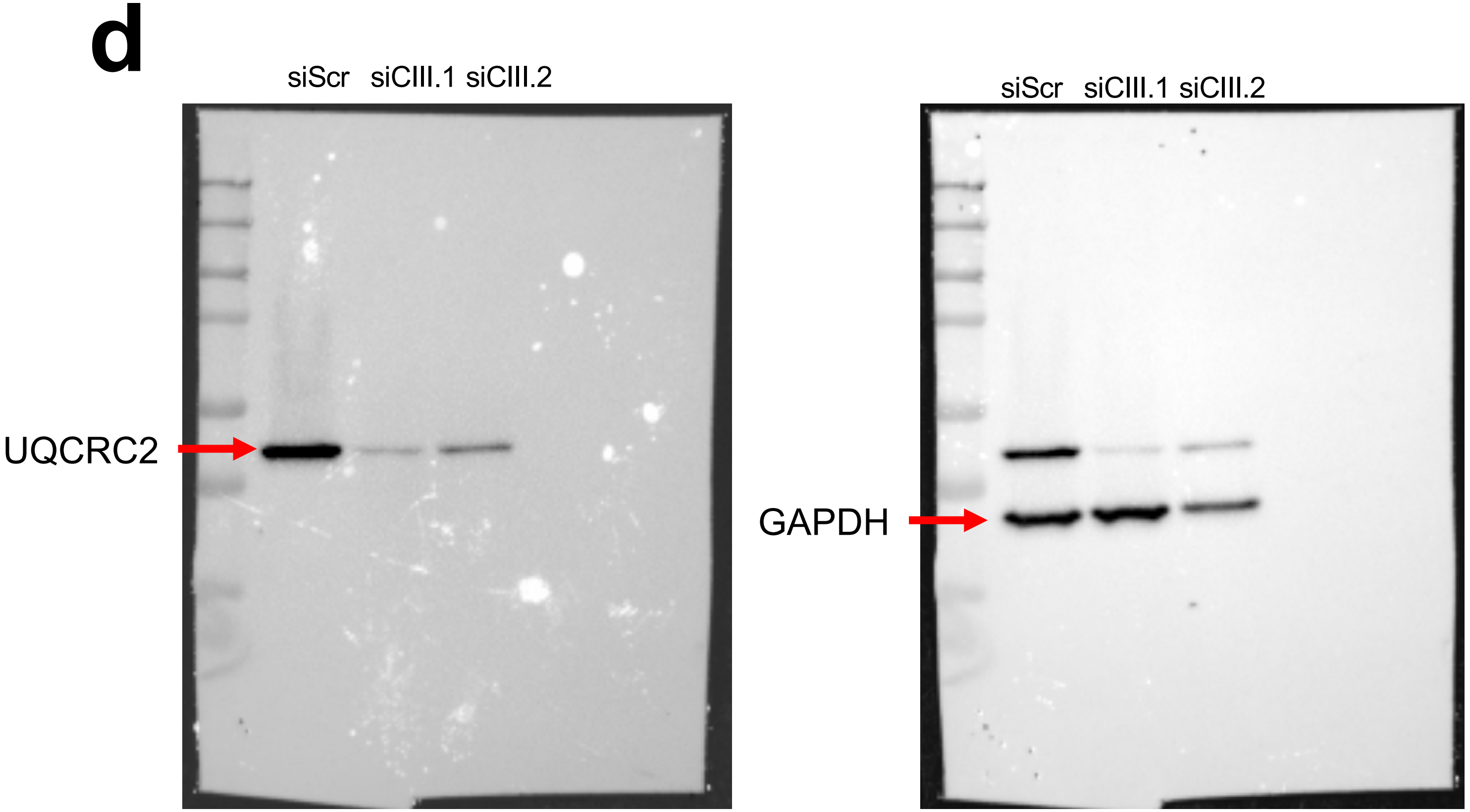

Figure 4h,i,j

<u>Legend</u>
siScr: MCF7 siRNA Scrambled
siCIII.1: MCF7 siRNA *UQCRC2* (#1)

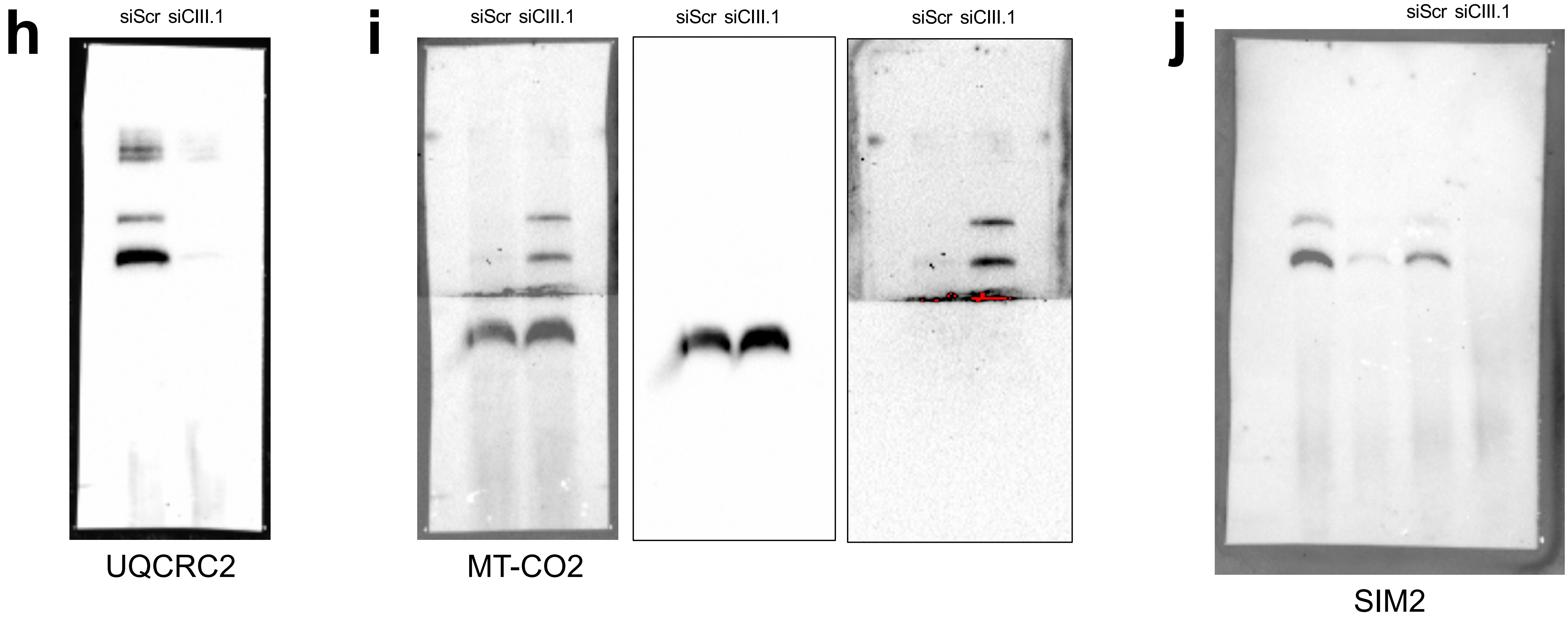

Figure 4k

Legend
siScr: MCF7 siRNA Scrambled
siCIII.1: MCF7 siRNA *UQCRC2* (#1)

k

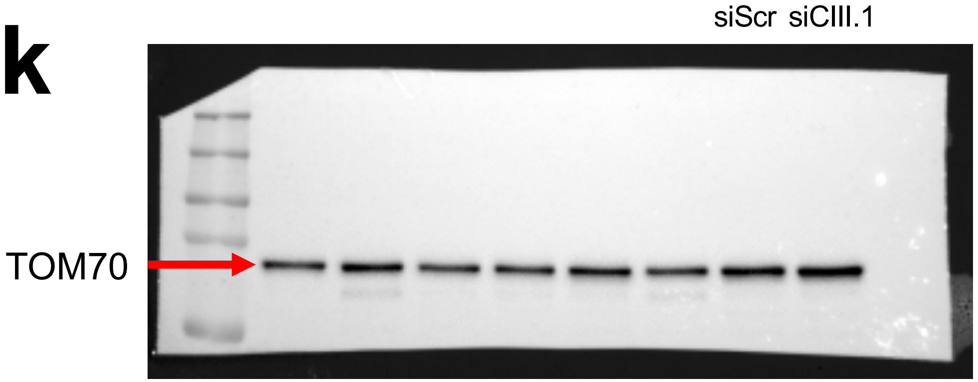

Figure 5k

Legend
M7: MCF7
MKO: MCF7 *SIM2KO*
SE: SUM159 *Empty*
SO: SUM159 *SIM2s-FLAG*

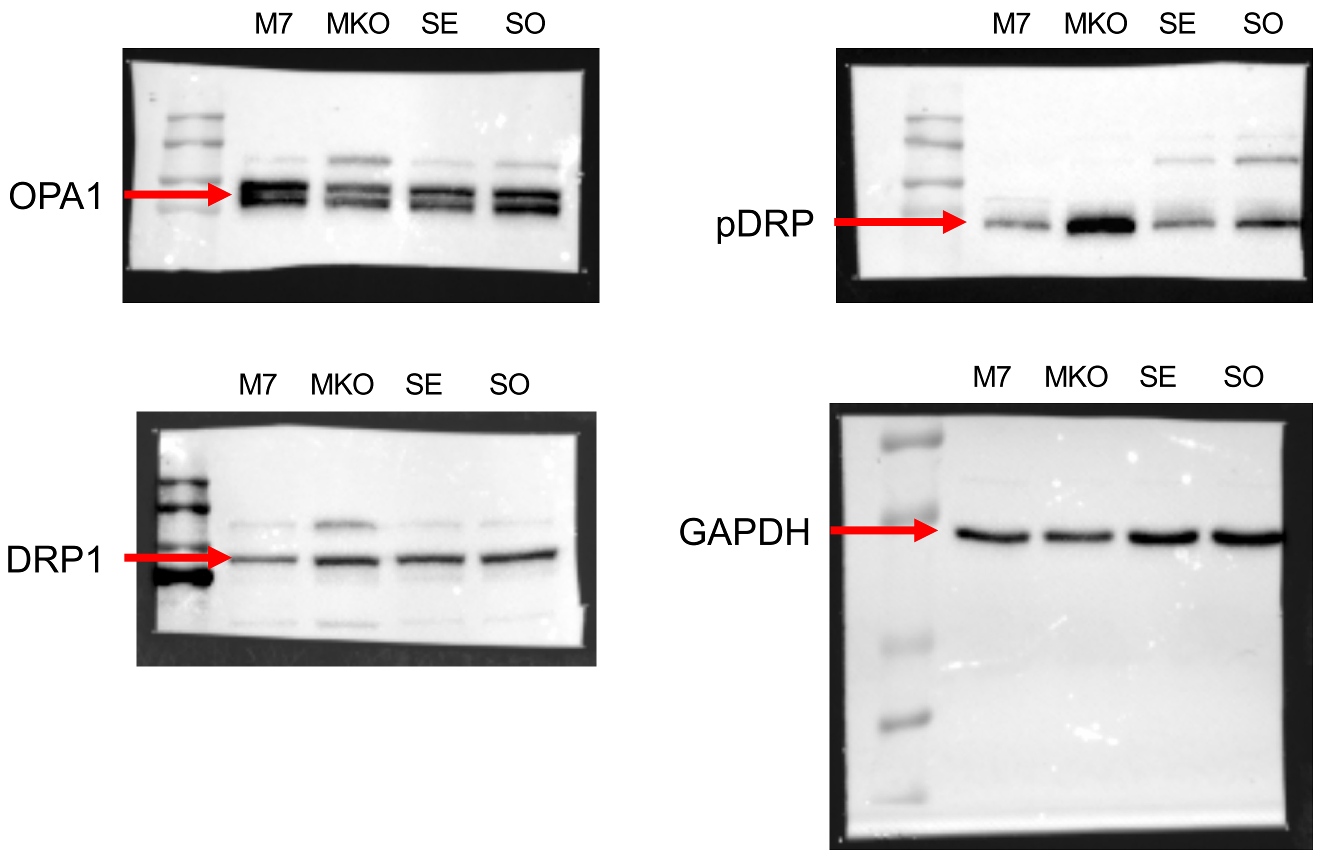

Figure 6g

Legend
M7: MCF7
MKO: MCF7 *SIM2KO*

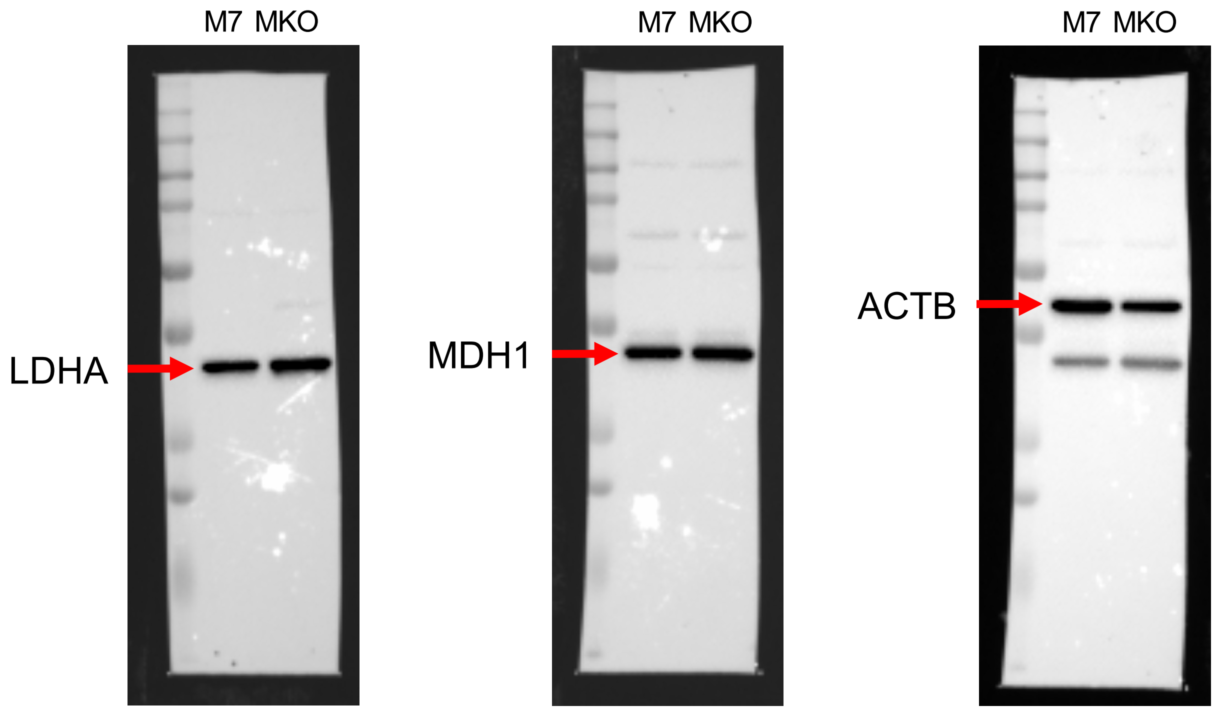

Supplement: Supplementary file 1 — Supplemental Material [file 12276_2023_996_MOESM1_ESM.pdf]
